# Teacher professional development for disability inclusion in low‐ and middle‐income Asia‐Pacific countries: An evidence and gap map

**DOI:** 10.1002/cl2.1287

**Published:** 2022-11-25

**Authors:** Syeda Kashfee Ahmed, David Jeffries, Anannya Chakraborty, Toby Carslake, Petra Lietz, Budiarti Rahayu, David Armstrong, Amit Kaushik, Kris Sundarsagar

**Affiliations:** ^1^ Australian Council for Educational Research Adelaide South Australia Australia; ^2^ Australian Council for Educational Research New Delhi India; ^3^ Australian Council for Educational Research Jakarta Indonesia; ^4^ RMIT University School of Education Melbourne Australia; ^5^ Australian Council for Educational Research Kuala Lumpur Malaysia

## Abstract

**Background:**

In the Asia‐Pacific region, around one‐third of the children who are out‐of‐school have a disability and given that teacher readiness and capability are key contributors for inclusive education, it is high time for a mapping of disability inclusive teacher professional development (TPD) interventions in this region.

**Objectives:**

The key objective of this evidence and gap map (EGM) is to locate evidence on interventions for in‐service TPD focussing on education for the inclusion of students with a disability in low‐ and middle‐income countries (LMICs) in the Asia‐Pacific region.

**Search Methods:**

A broad range of bibliographic databases and repositories were searched electronically to identify the evidence published between January 2000 and December 2021. Key search platforms included the British Education Index (BEI), Education Research Complete (ERC), Education Resources Information Center (ERIC), SCOPUS, 3ie Development Evidence Portal (Evidence Hub) and the Campbell Collaborations Systematic Reviews and EGMs portal (Better evidence for a better world). In addition, potential program evaluations/impact reports, reviews, case studies, and program descriptions/summaries were sought through ‘snowballing’ based on searching bibliographies and reference lists of papers located during the search process, as well as specific searches of relevant grey literature.

**Selection Criteria:**

To be eligible for inclusion, studies had to contain sufficient details about TPD interventions that support early childhood educators and kindergarten to Year 12 teachers to understand the needs of students with disabilities and aid them to create inclusive mainstream classrooms and/or provide improved support for students with disabilities in special education settings.

**Data Collection and Analysis:**

A total of 820 records were entered into the MS Excel file in which the entire data extraction process was managed. All records were screened against the predefined inclusion and exclusion criteria. Data were extracted independently by two reviewers and any differences were resolved through consultations. All included studies and their characteristics were extracted from the MS Excel file and uploaded to the ACER server in.csv file format. The interactive, online EGM is available here: https://datavis.acer.org/gem/disability-inclusion-TPD/.

**Main Results:**

Fifty studies from 16 countries out of the 41 LMICs in the Asia‐Pacific region were identified, whereby Thailand had the largest number of studies with evidence (7) followed by China, Vietnam, and India (5 each). Two main gaps in research about professional learning were identified. First, only three studies reported interventions aimed at supporting mental health among students with a disability. Second, no studies were found that reported on how teachers could support positive student behaviour. These gaps are important because research has persistently suggested that experiencing disability is an important risk factor for young people developing mental health conditions.

**Authors' Conclusions:**

This report illustrates the critical value of evaluating and publishing evidence from disability inclusive TPD interventions in LMICs, including any that are ongoing, or are components of highly resource intensive large‐scale education sector programs.

## 1 PLAIN LANGUAGE SUMMARY

### 1.1 Evidence and gap map finds 50 studies on teacher training for the inclusion of students with disabilities in low‐ and middle‐income Asia‐Pacific countries

Only 16 out of 41 countries report evidence on in‐service teacher professional development for disability inclusion in low‐ and middle‐income countries (LMICs) of the Asia‐Pacific region. These LMICs are still transitioning from segregated schooling to inclusive education. A majority of the identified interventions focus on changing teacher attitudes towards the inclusion of students with disabilities and understanding different forms of disabilities.

### 1.2 What is this EGM about?

More than 1 billion people live with disabilities, 80% of them in LMICs. While it is widely recognised that teacher readiness and capability are key contributors to a successful transition towards disability‐inclusive education, in‐service teacher professional development for disability inclusion remains an under‐researched area.

This evidence and gap map will help governments, schools, and policymakers to identify areas where there is sufficient evidence and areas where more evidence is needed. The EGM will assist agencies in deciding where to channel their resources, to:

- support interventions with a greater evidence base
- improve evidence collection where the evidence base is weak
- re‐assess support for current interventions.

### 1.3 What is the aim of this EGM?

The aim of this EGM is to identify evidence on interventions focused on in‐service teacher professional development for the inclusion of students with disabilities. The EGM considers early years to Year 12 education in LMICs in the Asia‐Pacific region.

### 1.4 What studies are included?

The 50 studies included in this EGM were published between January 2000 and December 2021. Most (29 studies) were published in the last five years. The studies vary greatly in their methods: a few have experimental designs and many use observational techniques for data collection.

### 1.5 What are the main findings of this EGM?

The included studies are unequally distributed across the intervention and outcome categories of the EGM. A significant number of interventions focus on changing teacher attitudes and understanding of disability, as many of these countries are in the early stages of the inclusive education agenda.

Only three studies discuss interventions for supporting mental health amongst students with a disability. One study reports an intervention to support students with physical disabilities where the teachers received training on mobility disability, as part of a larger teacher development programme.

Almost half of the 50 included studies are in mainstream school settings. None of the interventions identified support students with disabilities during emergency or crisis situations. This should be a key focus in light of the current pandemic and widespread environmental disasters.

### 1.6 What do the findings of the map mean?

The evidence collated here is unevenly distributed and there is room for more studies in this space. The EGM highlights the following needs:

- Uptake of primary research using robust methods to measure intervention effectiveness and impact
- Interventions designed to support school mental‐health and psychosocial wellbeing of students with a disability (SWD)
- Support for education systems’ efforts on evidence synthesis through regional alliances and the formation of evidence hubs.

### 1.7 How up‐to‐date is this EGM?

The authors searched for studies published up to December 2021.

## 2 BACKGROUND

### 2.1 Introduction

#### 2.1.1 The problem, condition or issue

The United Nations’ 2030 Agenda for Sustainable Development calls for ‘inclusive and equitable quality education that promotes lifelong learning opportunities for all’ (UNESCO, 2020, p. 1). In particular, the Sustainable Development Goal target 4.5 which focuses on inclusive education (IE) for the vulnerable and children with disabilities, receives a strategic mention (UNESCO, 2016). According to General Comment No. 4 (Article 24) of the CRPD:

…some groups are more at risk of exclusion from education than others, such as: persons with intellectual disabilities or multiple disabilities, persons who are deafblind, persons with autism or persons with disabilities in humanitarian emergencies (CRPD, 2016, p. 3).

Advocates of educational inclusion call for a fundamental reform of schools and the modernisation of education systems (Azorín & Ainscow, 2020). An important clarification by UNICEF on how to implement inclusion in schools highlights the transformative role of inclusive education: ‘… making sure that teaching and the curriculum, school buildings, classrooms, play areas, transport and toilets are appropriate for all children at all levels’, thus emphasising that ‘inclusive education means all children learn together in the same schools’ (UNICEF, 2017, p. 1). Similarly, UNESCO's ‘concept note’ for the 2020 Global Education Monitoring Report on Inclusion and Education indicates that the definition of inclusion has changed over the years from students with disabilities requiring separate classes and specialised teaching techniques to ‘a broader view, focused on ensuring that all students and students with disabilities are included in mainstream classes’ (UNESCO, 2018, p. 4).

##### Education for the inclusion of students with a disability

Disability is a formal diagnostic label for the difficulties with everyday life faced by an individual (Armstrong & Squires, 2014) and has been defined as ‘a complex and multidimensional issue’ (Commonwealth of Australia, DFAT, 2016, p. 7). However, the focus is primarily on impairment, which captures the impact of a disability on the daily life of a student. An emphasis on impact rather than on diagnostic classification has been recommended by researchers as it relates to the supports and possible interventions necessary to facilitate inclusion (Armstrong & Squires, 2014).

Inclusion of students with disabilities has many advantages for all students, and ‘promotes cooperative, collaborative activities and increases positive attitudes towards disability, reducing stigma and discrimination and leading to inclusive societies’ (DFAT, 2019, p. 4). Prior studies have noted significant benefits of IE for children with disabilities, particularly children with severe, complex, or multiple disabilities (Hunt, 2020; Katz & Mirenda, 2002). Studies have pointed out the advantages of IE for students with disabilities in terms of improved learning outcomes, including academic gains, improved communication and motor skills, higher social engagement (Hunt, 2019), stronger reading and mathematics skills, increased attendance rates, fewer behavioural problems, better social connections, and improved transition to post‐secondary level (Hehir et al., 2016).

Research over the last two decades suggests how a range of factors operating at different levels affect the implementation of education for the inclusion of students with a disability. Thus, the implementation of policy initiatives at state or local level intended to promote social inclusion (Bills et al., 2020), school leaders’ commitment to inclusion (Ainscow, 2020) as well as teacher practices in the classroom (Finkelstein et al., 2019), have emerged as important factors. In addition, attitudinal barriers by teachers responsible for implementing education for the inclusion of students with a disability have emerged as a reoccurring theme and found to be essential for the effective implementation of inclusion (Moberg & Savolainen, 2003; Savolainen et al., 2020; Van Mieghem et al., 2020).

These attitudinal barriers need to be examined from a broader perspective. While teachers are an essential component of education systems in general, this particularly applies in LMICs where infrastructure and resources tend to be scarce, leading to additional challenges for education for the inclusion of students with a disability (DFAT, 2019; UNESCO, 2020). More specifically, the GEM 2020 *Inclusion and Education* report describes barriers such as large pupil to teacher ratios, a lack of education support, weak professional teacher networks and a lack of autonomy over content (UNESCO, 2020).

Evidence from LMICs also suggests that teachers often lack the knowledge and skills for recognising and supporting students with disabilities (Ghimire, 2017; Kutcher et al., 2013; Shari & Vranda, 2015). Moreover, a lack of encouragement for teachers (e.g., a lack of increased pay or improved work conditions) (Muwana & Ostrosky, 2014) and widespread teacher‐centred methods of instruction (Arbeiter & Hartley, 2002) further impede the implementation of inclusion in these contexts (Wapling, 2016). Examples from Cambodia and India illustrate these issues where classroom practices were dependent on more traditional, less interactive teaching methods, in addition to overcrowded classrooms, scarce teaching resources and overambitious curricula, which made it harder for teachers to facilitate instruction with a focus on individual students or small groups of students (Singal et al., 2018; Song, 2015).

##### Issues affecting education for the inclusion of students with a disability in the Asia‐Pacific region

In the Asia‐Pacific region, around one‐third of the children who are out‐of‐school have a disability (Modern et al., 2010). This indicates the need for appropriate education services that support the learning goals of children with disabilities to unleash their full potential (DFAT, 2015b). Additionally, 52.7% of students with disabilities drop out of secondary schools, mostly from mainstream schools (UNESCAP, 2019). According to UNICEF, 43 million children with disabilities live in East Asia and the Pacific and the exclusion of these children from school is widespread in every country in this region (2021). The 2015 attendance data from 21 education systems in the Asia‐Pacific region suggests that only 19% of children (on average) with disabilities attended special primary schools (UN, 2018). Often, children with disabilities dropped out because of the financial burden on their families or contextual challenges (UN, 2018). One of Australia's key responses to this challenge has been through the provision of funds to ‘improve the accessibility to and quality of education for people with disabilities through policy dialogue, teacher training, curriculum development and education infrastructure’ (DFAT, 2015b, p. 10) in the region. Yet, the transition from segregated schooling to inclusive education and teacher education reforms has been sluggish (Forlin, 2010; Wu‐Tien et al., 2008).

In Southeast Asia, teachers and pre‐service teachers mostly hold negative attitudes towards IE for students with disabilities (Forlin et al., 2007, 2009; Sharma et al., 2006). Some reasons for this include a ‘lack of policy enforcement, lack of resources, lack of trained personnel, inflexible school system, merit‐oriented educational system, and also, societal attitude towards disability’ (Bradshaw & Mundia, 2005; as cited in Low et al., 2018, p. 237). The influence of community/societal attitudes and beliefs on the beliefs and attitudes of teachers cannot be ignored. Collectively, studies by Hopf et al. (2017), Kuzma et al. (2016) and Kamenopoulou and Dukpa (2018), in Fiji, Papua New Guinea, and Bhutan, respectively, highlight several attitudinal barriers to the effective implementation of education for the inclusion of students with a disability in these LMICs.

Even in some high‐income locations in the region, such as Hong Kong and Singapore, high parenting pressure within some communities can lead parents to internalise social stigma (Mak & Kwok, 2010; Wong et al., 2015) which results in keeping their children with disabilities at home.

In most schools in this region, educational segregation of students with disabilities is accepted, and teachers largely believe it is appropriate for children with disabilities to be taught by special education teachers (Lee & Low, 2013; Low et al., 2018). In Malaysia, for instance, ‘it is expected that the preservice teachers in the regular subject areas would not perceive that it is their responsibility to teach students with disabilities, whilst the special education teachers would perceive teaching students with disabilities to be their distinct responsibilities’ (Low et al., 2018, p. 238).

Besides, mainstream teachers may not be using teaching‐learning practices suitable for inclusive classrooms and ‘there is widespread acknowledgement that pedagogy is out of sync with the demands and challenges of the inclusive educational environment’ (Rieser, 2013, p. 68). This is enhanced by the reality that teaching and learning in the Asia‐Pacific region is often driven by assessment results, creating a conflict between high achievement scores and inclusion (Forlin, 2010). Some mainstream teachers may even be pushing out students with disabilities from their classrooms because they are not sufficiently skilled to manage inclusive classrooms (Nes et al., 2017).

Also, research has shown that teachers require in‐depth training to learn how to effectively implement assistive technologies (Blossom Cygnet et al., 2019; McMillan & Renzaglia, 2014) that help students with disabilities to perform tasks and improve their functional capacity to participate in everyday activities.

Lately, this transition to education for the inclusion of students with a disability has gained momentum in the region and it is widely acknowledged that funding effective teacher professional development (TPD) programmes has the potential to create a profound impact on the wellbeing and school outcomes of students with disabilities. In this context, Australia is one of the key partners in supporting the education of students with disabilities by providing funds to the development of teacher training programmes in the region (DFAT, 2015b).

Against this background, an Evidence Gap Map (EGM) of TPD interventions supporting the inclusion of students with disabilities is useful and timely.

#### 2.1.2 Scope of the EGM

TPD programs are the key to transitioning to education for the inclusion of students with a disability (CRPD, 2016). Since disabilities are complex, with changing definitions and thresholds for identification, teachers require regular professional learning to support education for the inclusion of students with a disability (Forlin & Sin, 2010). One recent study from transnational and cross‐sector perspectives has suggested that to enable inclusion, teachers ‘require professional learning that is collaborative, interprofessional, and acknowledges that the challenges they face are multifaceted’ (Beaton et al., 2021, p. 1). Although globally, inclusive education is accepted as the most suitable approach to ensure universality and non‐discrimination in the right to education, many countries and especially resource poor LMICs, still have students with disabilities learning in a range of settings including special schools, integration classes in regular schools as well as in inclusive classrooms. Preventing this dilution of inclusion is the purpose of UNICEF's statement (UNICEF, 2017) which explicitly calls for special schools to cease because they are incompatible with inclusion.

This current EGM focuses on LMICs in the Asia‐Pacific region, covering 41 education systems as specified by the Australian Government Department of Foreign Affairs and Trade's (DFAT) list of economically developing countries (DFAT, 2018). Many of these LMICs have education systems which need support in different areas including infrastructure, school governance reforms, teacher education, teacher recruitment and management, and learning assessment systems. Others are only starting their journey towards education for the inclusion of students with a disability. Thus, for example, Fiji established the *2016 Policy on Special and Inclusive Education* which documents the need for preparing teachers for screening and referring students with disabilities (Ministry of Education Heritage and Arts, 2016; UNESCO, 2020), while in Gujarat, a state in western India, health and education departments collaboratively developed a training program for the early identification of children with learning disorders such as dyslexia (Shastri, 2019; UNESCO, 2020). Some other countries are yet to establish policies which would result in the delivery of professional development opportunities for inclusion and supporting children with disabilities (UNESCO, 2020). For instance, in Bangladesh, teachers have reported an absence of professional development programs (both pre‐ and in‐service) for supporting children with disabilities (Rahaman, 2017). International data from the OECD Teaching and learning International Survey (TALIS) 2018 shows that even with 52% of teachers in primary education participating in TPD on teaching students with special needs in the 12 months before completing the survey, around 28% of teachers still reported a high need for it (OECD, 2021). Besides, the UNESCO GEM report notes a high demand from teachers in many middle‐ and high‐income countries for TPD programs that support teaching children with disabilities (UNESCO, 2020).

While both pre‐ and in‐service teacher development programs are needed to support teachers in transitioning to an inclusive education system, the current EGM compiles information on **in‐service TPD interventions** only for the following reasons:

- In‐service programs can have a more immediate impact on the inclusion of students with disabilities in classrooms as they focus on practices and attitudes of current teachers.
- In‐service learning programs are usually practice‐oriented with suggestions of how to make pedagogical practices more inclusive.
- Pre‐service education does not always equip teachers with competencies required to deal with everyday classroom challenges (Forlin, 2010). ‘Whether newly qualified teachers (NQTs) consider that they are sufficiently prepared to teach students with SEN in regular classes continues to be a cause for concern…’ (Forlin, 2010, p. 180).
- Many teachers who have been in the profession for decades may not have received any formal training on education for the inclusion of students with a disability. A study examining the skills of regular primary and secondary school teachers in Delhi in India found that nearly 70% of regular schoolteachers did not get training in special education and lacked experience of working with children with special needs (Das et al., 2013).

In summary, while most EGMs tend to have a broader scope, given the importance of the issue in this region, the authors are focused on synthesising evidence of TPD interventions for education for the inclusion of students with a disability in the Asia‐Pacific LMICs only. This work and its scope have been supported by discussions with key funders and education experts in the region—such as DFAT and Australian Council *for* Educational Research (ACER) offices in India, Indonesia, and Malaysia. Stakeholders agreed on the need to have more information about the TPD interventions focused on education for the inclusion of students with a disability in this region rather than the full spectrum of TPD programs out there, since they do not tell the regional policymakers much about teachers’ preparedness and needs on supporting disability inclusive education.

#### 2.1.3 Why it is important to develop the EGM

The Asia‐Pacific region is frequently affected by a range of natural disasters that impact the education of all children (UNESCAP, 2019) and that makes it particularly difficult to provide quality education to children with disabilities when they occur (INEE, 2009). The recent pandemic and environmental disasters such as widespread floods, have created additional obstacles to the transition to education for the inclusion of students with a disability in most LMICs (World Bank, 2020). The Christian Blind Mission (CBM) Australia for UNICEF's East Asia and the Pacific Regional Office and UNICEF Australia emphasises a further need to support teachers with training on education for the inclusion of students with a disability particularly due to the added health and wellbeing complexities owing to this pandemic and other recent climate change challenges (UNICEF, 2020), as well as advocates the provision of additional TPD, support, and mentoring for empowering teachers (UNICEF, 2020).

Therefore, a mapping of disability inclusive TPD interventions in this region is valuable and timely to gain more insights into the current situation and future needs for this sector. The content focus suggested for this EGM helps to keep this evidence synthesis manageable, appropriate, and relevant for interested funders and implementing agencies, who primarily support education for the inclusion of students with a disability in the LMICs of the Asia‐Pacific region. The geographical focus means a greater potential for TPDs to be replicated or adapted as countries in the region share some common cultures, backgrounds, and histories.

## 3 OBJECTIVES

As researchers and policy makers are often unaware of the extent of the evidence base, an evidence map (EGM) is a way of making explicit and accessible different interventions on a certain topic in a specified geographic area, to ‘guide users to available relevant evidence to inform intervention and design and implementation’ (White et al., 2020, p. 3).

The key objective of this EGM is to locate evidence on interventions focused on teacher professional learning and development (TPD) for the education for the inclusion of students with a disability in LMICs in the Asia‐Pacific region. As such, it illustrates different levels of evidence for TPD interventions as well as where there is no evidence (i.e., gaps). In other words, the EGM aims to empower agencies to better target resources by:

- Supporting interventions with a greater evidence base
- Supporting evidence collection in areas where the evidence base is weak
- Re‐assessing support for current interventions in light of the available evidence (White et al., 2020).

### 3.1 Existing EGMs and/or relevant systematic reviews

An earlier critical review by Waitoller and Artiles (2013) examined research evidence from professional development studies focused on inclusive education and found six types of TPD for inclusive education: action research, on‐site training, university classes, professional development schools, online courses, and a special educator's weekly newsletter on how to include children with disabilities. However, this review could not locate any systematic reviews on TPD for inclusive education and most reviews on TPD focused on studies conducted in Australia the UK, and the US.

A recent meta review by Van Mieghem et al. (2020) identifies four substantive aspects of the implementation of IE: (1) attitudes towards IE; (2) teachers’ professional development fostering IE; (3) practices enhancing IE and (4) participation of students with SEN. Van Mieghem and colleagues identified four reviews that highlights the TPD for inclusion theme: Kurniawati et al. (2014), Loreman (2014), Qi and Ha (2012), Roberts and Simpson (2016). A key finding in this area is that TPD is more effective when it focuses on specific student needs or disabilities, rather than on inclusion generally (Kurniawati et al., 2014), while a focus on specific teachers’ concerns and their teaching context is the most helpful in encouraging change in teachers’ practice (Kurniawati et al., 2014; Qi & Ha, 2012; Roberts & Simpson, 2016). Van Mieghem et al. (2020) concludes that TPD on evidence‐informed inclusive practices leading to successful teacher experiences is the cornerstone for the implementation of inclusive education.

A current EGM on disability interventions (Saran et al., 2020) illustrates various initiatives for improving health, education, livelihood, social issues, empowerment and advocacy and governance for people with disabilities. However, this review reports only a single study on in‐service TPD in Kenya (Carew et al., 2019).

A key point to note is that most research in this space focuses on evidence from interventions that attempt to improve skills in the students with disabilities ‘rather than addressing institutional or environmental barriers, which are often the key focus of disability‐inclusive development’ (Kuper et al., 2020, p. 2). For instance, an earlier review by Bakhshi et al. (2013) analysed programs that increased the accessibility to education for children with disability aged between 4 and 18 years across economically developed and developing countries but did not include any TPD intervention.

A recent Rapid Evidence Assessment (REA) by Kuper et al. (2018) of What Works to Improve Educational Outcomes for People with Disabilities in Low‐ and Middle‐Income Countries focussed on interventions to improve educational outcomes for people with disabilities in LMICs, which reported a few TPD interventions (Carew et al., 2019; DeVries et al., 2018; Martin et al., 2001) from China, Kenya and Uganda, respectively.

In summary, prior research identifies teacher readiness (Van Mieghem et al., 2020) as a major factor for a successful transition towards education for the inclusion of students with a disability while relevant work summarised here either does not cover TPD or countries outside the Asia‐Pacific region. Hence, this EGM is timely and highly focused to provide a useful information base for targeted stakeholders.

## 4 METHODS

The published protocol covered the conceptual model of the EGM, and the EGM framework, in addition to defining the methods, selection criteria and the strategy for data collection and analysis. These are briefly discussed in the following sections.

### 4.1 Evidence and gap map (EGM): Definition and purpose

EGMs ‘are a systematic evidence synthesis product’ (White et al., 2020, p. 1) intended to guide researchers and policymakers to high quality evidence to identify research gaps, inform research priority setting, and support evidence‐based decision making (Katz et al., 2003; Saran & White, 2018). Over time, different agencies have defined such evidence maps in different ways and used different approaches to generating such maps. However, Saran and White (2018, p. 9) discuss key components that should be present in any evidence maps which include the following:

- Systematic approach
- The type of evidence included
- The content of the map
- The structure of the map
- Graphical display
- Accompanying description of map
- Intended users of the map.

Results from such evidence syntheses are valued by development partners who prefer to make investment decisions which are based on high quality evidence (e.g., DFAT, 2015a, 2015b; DFID, 2013; Jones, 2012; USAID, 2019). In recent years, such maps have gained popularity, particularly in the international development field. Thus, for example, a recent ‘map of maps’ commissioned for international development interventions (Phillips et al., 2017) reported as many as 73 maps (Saran & White, 2018). While most evidence maps are broader in scope a few are quite focused (e.g., Bakrania et al., 2018; Robinson & Rust‐Smith, 2017).

**Figure 1:**
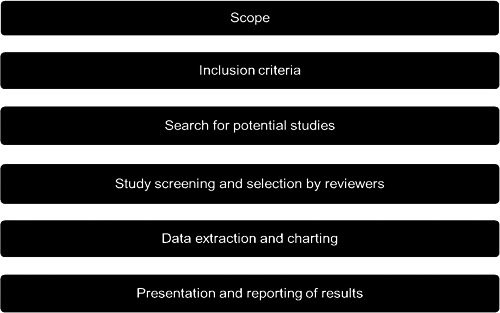
Steps for producing an evidence and gap map. Adapted from Campbell Collaboration (2020), https://www.campbellcollaboration.org/evidence-gap-maps.html; Saran and White (2018), https://onlinelibrary.wiley.com/doi/epdf/10.4073/cmdp.2018.2

Figure 1 outlines the process involved in conducting this EGM which is based on the methodological steps suggested by the Campbell Collaboration (White et al., 2020). This method involves (a) the development of the review's scope, (b) the setting of inclusion criteria, (c) searching for and identifying relevant studies, (d) screening and assessing studies for inclusion, (e) extracting and charting the data and (f) presenting and reporting the results.

In line with the Campbell EGM guidance that critical appraisal of all included studies is desirable but not mandatory (Saran & White, 2018; White et al., 2020), therefore this step was taken out in the interest of producing the EGM within a strict timeframe, which is shorter than what would have been required for a full‐sized systematic review. The search for this EGM was quite comprehensive and systematic, similar to a systematic review search. However, some of the more stringent search steps were not undertaken to enable this work to be completed within the planned timeframe. For instance, the search statement used for the current work, relied heavily on subject terms to provide a more specific search with more relevant results. In contrast, the search statement for a systematic review would have been broadened to rely less on subject terms and to consider more variations including proximity operators.

The data extraction step essentially follows the elements suggested by Saran and White (2018, p. 16) by charting the:

- Intervention categories
- Outcome categories
- Status of the study: completed or ongoing
- Geographical coverage of the study, where applicable
- Inclusion criteria of any included systematic reviews
- Primary study design.

Alongside this EGM report, a visual representation of the results, that is, interactive EGM has been published through the ACER data visualisation website.

### 4.2 Framework development and scope

#### 4.2.1 Stakeholder engagement

Advice from DFAT and CBM on an initial draft EGM proposal has been helpful for refining the direction of this work and the inclusion of practice‐based evidence. In addition to first scans of evidence emerging from initial topical searches, feedback from the following stakeholder engagements has further clarified the topic and scope of this EGM:

- Initial consultations with the GEM Centre Executives on the value of this work for ACER and its alignment with the GEM Centre's principles.
- Sharing of the initial study proposal with DFAT Education Section and their Disability Technical Partners Christian Blind Mission (CBM) Global Disability Inclusion Group during December 2019.
- Guidance on the scope and inclusion/exclusion criteria from subject experts—Dr David Armstrong, Editor, Journal of Research in Special Educational Needs (JORSEN) and Dr Jane Jarvis, Co‐chair, Research in Inclusive & Specialised Education (RISE), Flinders University.
- Presentation of the scope, methods, and initial findings at the Educational Research (Re) connecting Communities (ECER) 2020, online conference (in the Network 4: Inclusive Education forum), organised by the European Educational Research Association (EERA) during August 2020.

### 4.3 Conceptual framework

Research shows that the provision of high‐quality inclusive education is mainly influenced by teachers and their ability to support and acknowledge students’ heterogeneous needs (Gomendio, 2017; Moen, 2008; Schwab & Alnahdi, 2020). More specifically, TPD is particularly relevant in the context of resource‐scarce LMICs in the Asia–Pacific region where teachers empowered with the right skills through interventions for the inclusion of students with disability can have a significant impact on student outcomes (Chakraborty et al., 2019; UNESCO, 2017).

According to a model put forward by Finkelstein et al. (2019), inclusive teacher practice has five key aspects, namely instructional practice, organisational practice, socio/emotional practice, determining progress, and collaboration and teamwork. Teachers’ expectations and beliefs‐in‐action resulting from social, cultural, and political influences have a dominating effect on teaching and learning in inclusive classrooms (Florian & Rouse, 2001; Howes et al., 2009). Thus, disability inclusive TPD not only needs to focus on eliminating stigma associated with disabilities but also create awareness and understanding of these issues to empower teachers.

In addition, it is equally important for education systems to assist teachers in developing the capabilities and confidence necessary to be inclusive of students with disabilities. In a high‐quality education system, teachers are supported through educational policies that focus on teachers’ wellbeing and inclusion, pre‐service learning, and ongoing professional development (Darling‐Hammond & Cook‐Harvey, 2018).

**Figure 2:**
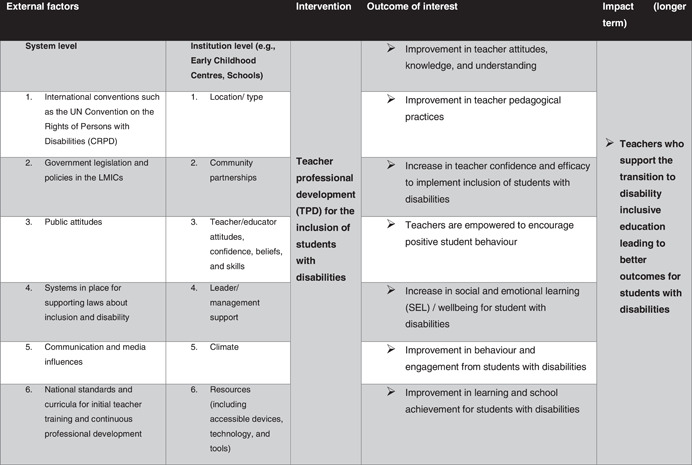
Conceptual framework of the EGM. This EGM framework informs the inclusion and exclusion criteria of the EGM. EGM, evidence and gap map.

Figure 2 provides a conceptual framework for exploring the disability inclusive TPD interventions and how these are linked to the outcomes of interest. This model does not represent a full theory of change of how specific interventions are meant to create impact. However, it does provide an overview of the relationships between external factors, interventions and outcomes and ultimate impact.

#### 4.3.1 Criteria for including and excluding studies

The criteria detailed in Table 1 have been considered when deciding whether to include or exclude a study/review in this EGM.

**Table 1:** Inclusion and exclusion criteria for the EGM

Selection criteria	Inclusion	Exclusion
Publication year	Studies published between January 2000 and December, 2021	Studies published before 2000
Publication status	Completed and on‐going	Planned
Study design	Primary studies (including quantitative, qualitative, or mixed methods), descriptive overview reports, systematic reviews and EGMs that are focussed on TPD for education for the inclusion of students with a disability	Reviews or EGMs that focus on TPD but are not focused on TPD for inclusion and disability
		Reviews or EGMs that include TPD studies for education for the inclusion of students with a disability from countries that are not listed under Asia‐Pacific region on the DFAT (2018) list of developing countries
Publication language	Studies/reviews published in English only,	Studies published in a language other than English. (Non‐English studies were excluded based the review teams’ own language skills, and resource needs (time and costs) that are required to involve professional translators.)
Population	In‐service teacher professional development (TPD) and/or professional learning programs	Interventions for pre‐service teachers during initial teacher education
Interventions	Programs that support teachers to understand the needs of students with disabilities	Programs that focus only on supporting teachers to accommodate other diverse groups, such as ethnic groups, migrant communities, children belonging to low‐socio‐economic status, refugees, and other minority groups
	Programs that support the integration and inclusion of students with disability in mainstream classrooms	
	Programs in special school settings that support students with disabilities	
	Evidence for practice‐based interventions (i.e., initiatives that have been undertaken/are being undertaken in LMICs in the region of interest) where there is sufficient information available about these in the grey literature searched	Practice‐based interventions (i.e., initiatives that have been undertaken/are being undertaken in LMICs in the region of interest) without sufficient information about the TPD program (or TPD component)
	Details should at least include:	For example:
	Intervention (or component) name that focuses on disability inclusive TPD Intervention categories Outcome categories Status of the program Geographical coverage Funding agency/implementing agency	Statements that are broad and vague, without providing details about a program (e.g., XYZ program has been running in the Pacific Islands and has supported students with disabilities through several initiatives, that also includes teacher professional training)
Context (geographic location and settings)	Interventions in low and middle‐income countries (LMICs) in the Asia‐Pacific region	Interventions in high‐income countries (HIs) in the Asia‐Pacific region or countries (including LMICs) from a different region.
	A relevant study found in a review which is from a country of interest will be included as a primary study – if the review covers interventions in other regions and countries it cannot be included as a review based on this inclusion criteria.	Interventions for teachers who are beyond school levels (such as faculties at tertiary education level institutions or vocational institutes).
	Interventions in early childhood settings including nurseries, playgroups, child‐care centres, or pre‐schools; and school settings including, K‐12 mainstream schools and/or special education schools.	
Intended outcomes	As specified in the EGM outcomes framework (See Table A1).	None
Quality	Not to be restricted based on any quality assessment.	None

Abbreviations: EGM, evidence and gap map; TPD, teacher professional development.

### 4.4 Dimensions

In line with the conceptual framework (Figure 2), this EGM has two main dimensions, with interventions shown in the rows and outcomes captured in the columns. As can be seen, interventions are categorised in two ways, namely by disability types and by special interest groups. Disability types include physical, mental, developmental and sensory disabilities. Special interest groups cover disability awareness, learning difficulties as well as specialised tools, approaches, and techniques for supporting Students with a Disability (SWD).

The outcomes of TPD interventions are categorised depending on whether those outcomes are aimed mainly at teachers or students. For teachers, the TPD interventions are categorised depending on whether they are aimed at changing teachers’ attitudes, knowledge and understanding of disability, pedagogical changes to support education for the inclusion of students with a disability in their classrooms, enabling positive student behaviour, and impacting their confidence and efficacy to implement education for the inclusion of students with a disability. In addition, some TPD interventions are also aimed at improving student outcomes, such as student learning and achievement, behaviour, and engagement in the classroom, and/or their social and emotional learning (SEL) and wellbeing. To be included in the review, the intervention must be aimed at—at least one—teacher outcome. Only then is it examined whether the intervention is also aimed at some form of student outcome (see further details under the heading ‘Outcome categories’ below). The interventions and outcomes are described in detail in the following sections.

#### 4.4.1 Intervention categories

As mentioned above, one way of categorising interventions in this review is by disability types which includes physical, mental, developmental, and sensory disabilities, based on formal diagnostic categorisations as specified below:

- A **physical impairment** affects the mobility or physical capacity of individuals. It may result, for example, from acquired brain injury, spinal cord injury, Spina bifida, Cerebral Palsy, Epilepsy (Aruma, 2019a).
- The World Network of Users and Survivors of Psychiatry suggested a change in the way persons with mental health disabilities are described and are to be referred to as persons with psychosocial disabilities (WNUSP, 2008). While we acknowledge the term psychosocial disability, for the purposes of this EGM **mental health condition** or another recognised classification, that is, **Developmental Disability (DD)** will be used.
- The American Psychiatric Association lists conditions such as Schizophrenia, Obsessive‐Compulsive, and Related Disorders as **mental health condition** (APA, 2020).
- **Developmental disabilities (DDs)** are defined by Zablotsky et al. (2019) as ‘a group of lifelong conditions due to an impairment in physical, learning, language, or behaviour areas’ and notes ‘Children diagnosed with developmental disabilities typically require services to address behavioural and developmental challenges’ (p. 144). While persons with ASD and Intellectual disability (ID) carry increased risk of developing a mental health issue (Matson & Williams, 2013) these are distinct, and therefore ASD and ID can be classified as a developmental disability (Zablotsky et al., 2019).
- A **sensory impairment**, on the other hand is associated with impediments to the senses, such as sight, hearing, smell, touch, and taste (Aruma, 2019b). DSM‐5 categorises communication disorders as a component of sensory disabilities comprising of Language Disorder, Speech Sound Disorder, Childhood‐Onset Fluency Disorder (Stuttering), and Social (Pragmatic) Communication Disorder (Paul, 2013). The American Speech‐Language‐Hearing Association (ASHA) also recognises hearing disorders as a communication disorder (ASHA, 1993).

The second way of categorising interventions is in terms of special interest groups. Thus, for example, the EGM includes interventions which support **disability awareness**, that is, knowledge and understanding of various disabilities and impairments, the impact that societal attitudes, inherent stigma, and discrimination, therefore encourage inclusion of SWD in classrooms. Others focus only on **learning difficulties**, such as, difficulties in learning to read (dyslexia), and write (dysgraphia) or other areas of learning, such as mathematics (dyscalculia), which do not fit precisely under the above types of disabilities/impairments but are vital for promoting inclusion in classrooms. Finally, some interventions focus on training or **teaching specialised tools, approaches, and techniques** (e.g., Functional behavioural assessment, cognitive strategy instruction, collaborative inquiry, and individual learning plans).

#### 4.4.2 Intended outcome categories

As the EGM is focussed on TPD, for interventions to be included they must have at least one of the following intended outcomes aimed at the teachers:

- Attitudes, knowledge and understanding
- Pedagogical practices
- Enabling positive student behaviour
- Confidence and efficacy to implement inclusion.

In addition, interventions may also have intended student level outcomes such as:

- Learning and achievement
- Behaviour and engagement
- Social and emotional learning/wellbeing.

These are discussed in further detail in Table A1.

#### 4.4.3 Types of study design

Since the main purpose of this review is to map the evidence for in‐service TPD for education for the inclusion of students with a disability in classrooms, a wide variety of study designs has been accepted if they added information on the topic of interest and helped to identify where evidence is currently available and where there are gaps.

This EGM therefore considers both qualitative and quantitative study designs (e.g., experimental, quasi‐experimental, before and after studies without control groups, descriptive studies, case studies, etc.) (see Table A1 for more details). The studies may follow any of these research methods or follow a mixed methods design if they meet the inclusion criteria. Additionally, any study with a TPD program impact summary/description which provides insights into the inclusion of students with disabilities for in‐service teachers in LMICs in the Asia‐Pacific region is eligible for inclusion if it meets all the criteria.

Any systematic review and/or EGMs focusing solely on TPD for education for the inclusion of students with a disability with studies only from LMICs in the Asia‐Pacific region are also eligible for inclusion.

However, we could not find any eligible systematic review for inclusion.

#### 4.4.4 Types of intervention/problem

Any type of TPD/learning program/intervention or in‐service training opportunity with the aim of creating or fostering disability inclusive classrooms for students with physical, mental, developmental, sensory, and or multiple or complex needs are eligible. Also, any TPD focused on supporting learning difficulties and specialised tools, approaches and techniques are eligible for inclusion as they support education for the inclusion of students with a disability outcome in classrooms.

For the practice‐based reviews in which only a subset of the interventions is eligible for inclusion in the map, only the relevant interventions (i.e., the relevant program component details) are included in the data extraction and mapping.

#### 4.4.5 Types of population (as applicable)

The population in focus are practicing teachers or special needs educators in early childhood centres or child‐care services, preschools, and schools who are working with children/students between the ages of 0 to 18 years.

The EGM also covers interventions for in‐service teachers and educators who work with students with special needs in mainstream schools or special schools or special education classrooms in mainstream schools.

#### 4.4.6 Types of outcome measures (as applicable)

##### Intended outcomes (prospective)

Included interventions have at least one teacher outcome and may also report student outcomes.

*Intended teacher outcomes*

- Attitudes, knowledge and understanding
- Pedagogical practices
- Enabling positive student behaviour
- Confidence and efficacy to implement inclusion.

*Intended student outcomes*

- Learning and achievement
- Behaviour and engagement
- Social and emotional learning/wellbeing.

See Table A1 for more details.

##### Unintended outcomes

Any potentially adverse or unintended outcomes of the interventions have been noted in this EGM report (see Table A2).

**Table 2:** Evidence by source

Evidence sources	Definition	Number of studies
Practice‐based	Practice‐based evidence in this EGM refers to other forms of reporting such as program reviews or case reports widely accepted by the international development agencies as valuable forms of evidence, some using mixed research methods but also includes many that could not be classified into a specific study design.	24
Systematic	Research studies and reviews that are published in peer reviewed journals, and dissertations, program impact evaluations are classed under ‘systematic data base searches’. These also include results from research evidence hubs and repositories (such as, 3ie Development Evidence Portal, Campbell Collaborations Systematic Reviews and EGMs portal, and Center on Knowledge Translation for Disability and Rehabilitation Research (KTDRR)).	26

#### 4.4.7 Other eligibility criteria

##### Types of location/situation (as applicable)

Only studies which focus on interventions in LMICs in the Asia‐Pacific region are included. The reason for this geographical focus is due to the Asia‐Pacific region being an area of strategic interest for many development partners (DPs) which value EGMs when making key investment/funding decisions (e.g., DFAT, 2015a, 2015b; DFID, 2013; Jones, 2012; USAID, 2019).

Besides, non‐English studies were excluded based the review teams’ own language skills, and resource needs (time and costs) that are required to involve professional translators.

##### Types of settings (as applicable)

The intervention could be set in any of the following:

- Early years settings including nurseries, playgroups, child‐care centres, or pre‐schools up to Year 2 (Ages 0–8)
- Mainstream schools (K‐12)
- Special education schools or classrooms.

##### Status of studies

The EGM covers both completed and on‐going studies which are presently in‐progress and have been documented in some form—for example, website descriptions of current programs, that are yet to undergo any formal evaluations if they included sufficient details.

### 4.5 Search methods and sources

An initial limited search of development partner portals was undertaken to scope several potentially relevant studies, including previous literature reviews and systematic reviews on in‐service teacher training for inclusion of students with disabilities in LMICs in the Asia‐Pacific region. Results of these searches has been used to further develop search terms.

A broad range of bibliographic databases and repositories have been searched electronically to locate the relevant evidence. Given, the time allocated for this EGM was shorter than that for a systematic review, the search statement was developed and adjusted accordingly. The information specialist relied heavily on subject terms to provide a more specific search with more relevant results within the timeframe provided. Also, keyword searches, including title and abstract were conducted on selected terms only relating to various disability terms—see terms and variations (*) not proceeded by SU—for example, see exclus* OR equit* OR inequit*OR marginali* OR ‘activity limitation’ OR ‘participation restriction’. The search platforms are listed below, along with the total number of search results for each:

- A+ Education (via Informit) (80 records)
- British Education Index and Education Research Complete (via EBSCO) (255 records)
- ERIC (via EBSCO) (293 records)
- SCOPUS (via Elsevier) (135 records)

Others (57 records)
  - Systematic review repositories (Campbell Collaborations Systematic Reviews and EGMs portal (Better evidence for a better world), 3ie Development Evidence Portal (Evidence Hub), Center on Knowledge Translation for Disability and Rehabilitation Research (KTDRR), EPPI (UCL—UK) Database of Educational Research, and Teacher Reference Centre (EBSCO)
  - Google scholar (first five pages of searches)
  - Development Partner Publication (DPP) portals—UNICEF, UNESCO, World Bank, USAID, Japan International Cooperation Agency (JICA), the Australian DFAT and the UK's Foreign, Commonwealth and Development Office (FCDO) (first three to five pages)

Moreover, potential program evaluations/impact reports, reviews, case studies, and program stories have been sought through ‘snowballing’ based on searching bibliographies and reference lists of articles located during the search process, as well as specific searches of relevant grey literature. Potential on‐going interventions that are identified through any of the above‐mentioned sources have been screened for inclusion in the EGM.

The searches for unpublished studies—and practice‐based studies (or implementation research reports on interventions)—have been conducted through the DPP portals. The DPP portal search was conducted during early 2022 and yielded a total of 27 papers of which 23 relevant papers included in this EGM. The final list of DPP portals from which studies were included are: Australian Aid, Health and Education Advice and Resource Team (HEART), International Assistance Mission (IAM), Save the Children, School‐to‐School International (STS), Oxfam India, Plan International Laos, UNICEF, UNESCO, Voluntary Service Overseas (VSO), and World Vision.

The systematic search was rerun by the study team during January 2022 as the initial search date was more than 6 months from the planned publication date. Results of the additional search have been thoroughly screened for potentially eligible studies.

Some practice‐based interventions were not included because they did not meet the strict inclusion criteria for this EGM. However, these have been included under the ‘Studies awaiting classification’ section of this report. These will be monitored and any emerging reports about these interventions could be included in updates of this EGM.

However, ongoing studies found through research database searches, which are past their registration cut‐off date or with uncertainty about their completion, or without sufficient details have not been included.

A sample search statement has been provided (see Supporting Information: Appendix C).

The protocol^1^ for this EGM has been published online (first published on 09 November 2021) and can be retrieved using the following link: https://onlinelibrary.wiley.com/doi/10.1002/cl2.1201.

### 4.6 Analysis and presentation

#### 4.6.1 Report structure

Briefly outline the structure of the report, specifying the tables and figures to be included in the report.

Abstract
Plain language summary
Background
  - Introduction
  - The problem, condition or issue
  - Scope of the EGM
  - Why it is important to develop the EGM

Objectives
  - Existing EGMs and/or relevant systematic reviews

Methods
  - EGM: definition and purpose
  - Framework development and scope
  - Stakeholder engagement
  - Conceptual framework
  - Dimensions
  - Search methods and sources
  - Analysis and presentation
  - Data collection and analysis

Results
  - Description of studies
  - Synthesis of included studies

Discussion
  - Summary of main results
  - Areas of major gaps in the evidence
  - Potential biases in the mapping process
  - Strengths
  - Limitations
  - Stakeholder Engagement throughout the EGM process

Authors' conclusions
  - Implications for research, practice and/or policy

Acknowledgements
Contributions of authors
Declarations of interest
  - Plans for updating the EGM

Differences between protocol and review
Additional tables
  - Table A1: Outcome categories for the EGM framework
  - Table A2: Data extraction table

References to studies
Sources of support
Appendices
  - Appendix A Link to online interactive EGM
  - Appendix B Studies awaiting classification
  - Appendix C Search statement

### 4.7 EGM presentation

**Figure 3:**
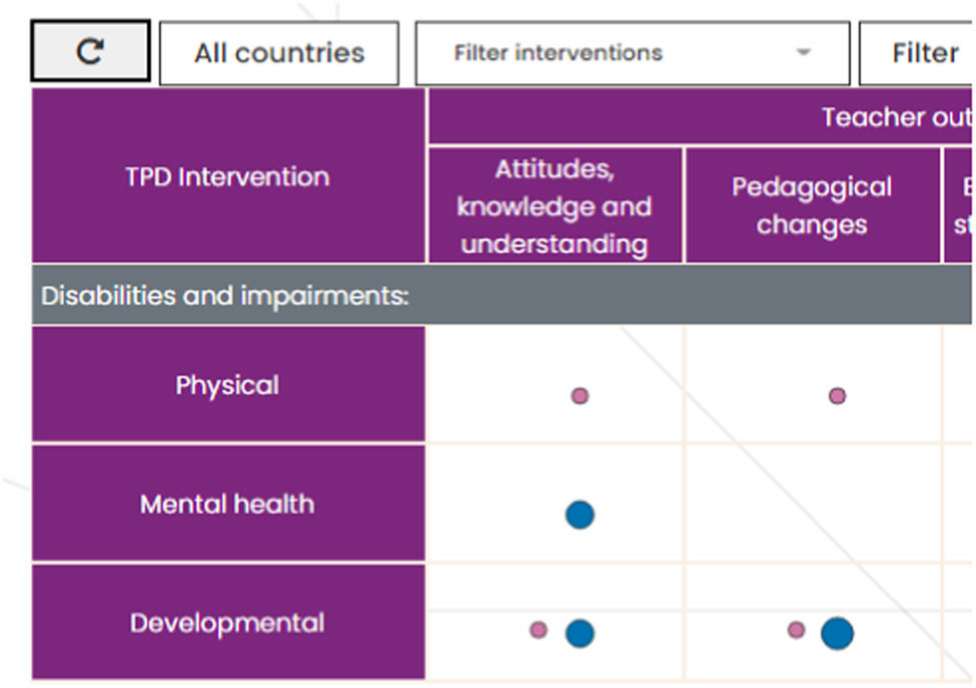
EGM cells. EGM, evidence and gap map.

In each cell (Figure 3), circles indicate whether evidence is available for intervention (rows) and outcome (columns) intersections. The size of the circle reflects the amount of available evidence, with the size increasing as the amount of evidence increases. Circles are also coloured pink and blue to which further separates the studies into those which report actual outcomes and those that intend to report outcomes.

**Figure 4:**
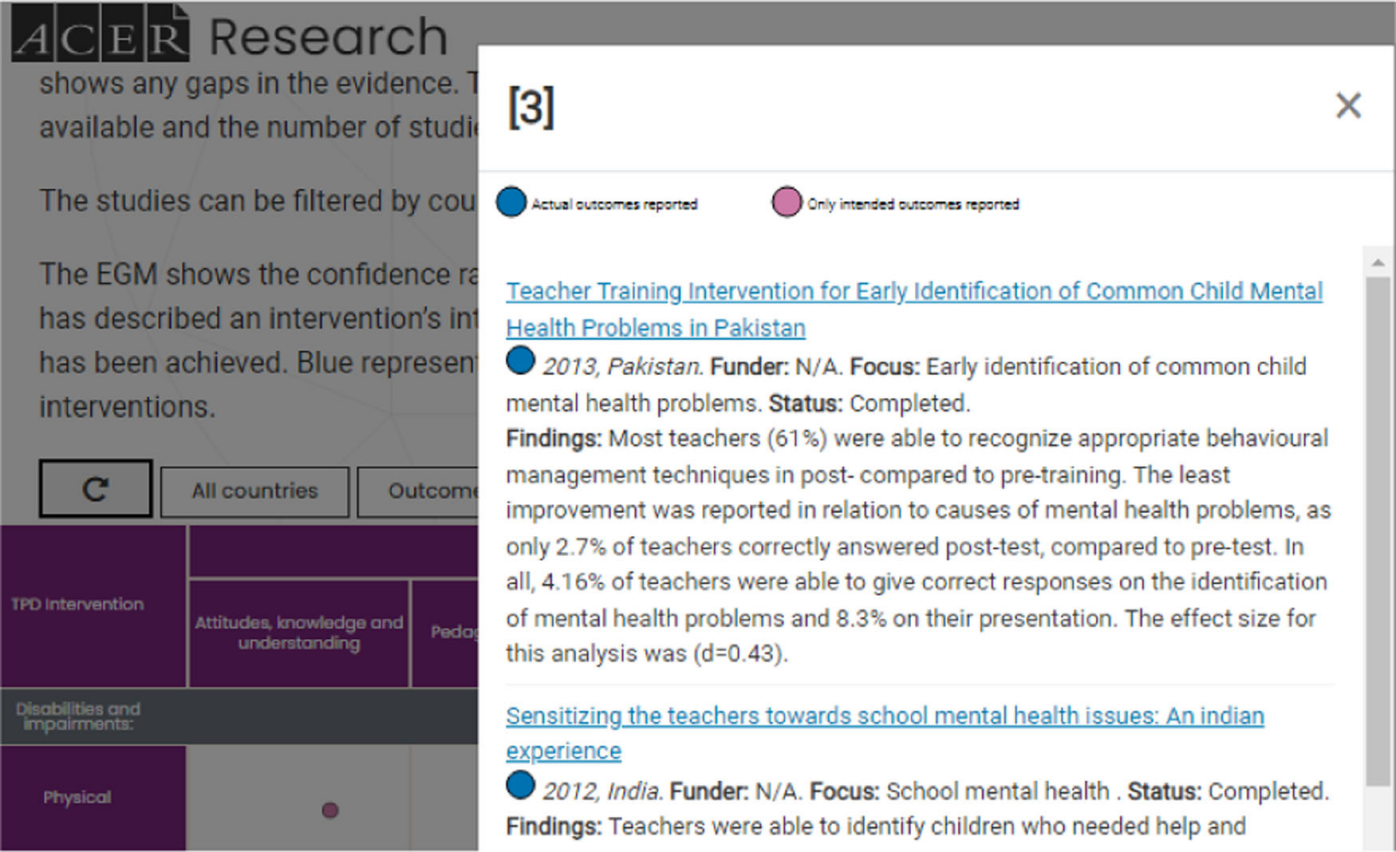
Study details pop‐up box

Hovering on a cell displays the total number of studies across both confidence categories (pink and blue) Clicking on a cell opens a pop‐up box (Figure 4) that provides additional information about studies in the intervention and outcome intersection.

The following details are included in the pop‐up box.

- Total number of studies included for the intervention and outcome intersection
- Title of study (with embedded hyperlink to full text journal article or report)
- Confidence rating
- Year of study publication
- Country where the intervention was conducted
- Funder
- Study focus
- Status
- Findings

#### 4.7.1 Filters for presentation

**Figure 5:**
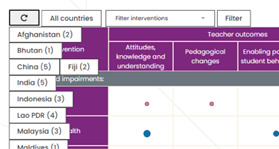
EGM country filter. EGM, evidence and gap map.

**Figure 6:**
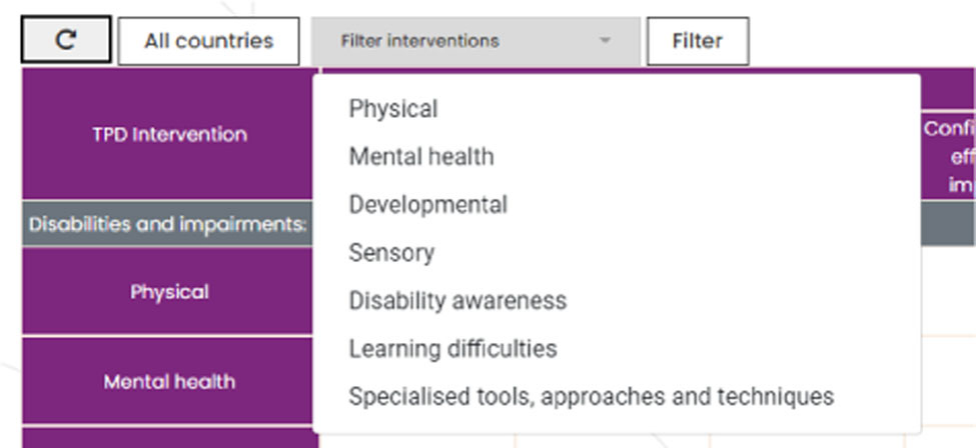
EGM intervention filter. EGM, evidence and gap map.

The EGM also includes filter functionality via two drop‐down menus. Studies displayed in the EGM can be filtered by country (Figure 5) and intervention (Figure 6).

Also included with the EGM site is a geographic map which displays all DFAT Asia‐Pacific countries. For countries that have included studies, the red circle (size increases as evidence increases) can be clicked to display further information about the studies carried out in that country.

The following details are included in the pop‐up box for the geographic map.

- Total number of studies included for the intervention‐outcome intersection
- Title of study (with embedded hyperlink to full text journal article or report)
- Confidence rating
- Year of study publication
- Funder
- Study focus
- Status
- Findings.

#### 4.7.2 Dependency

For this EGM, each study about an intervention has been considered as the unit of analysis for primary studies. Therefore, if multiple studies report on the same intervention all these individual studies are included as separate pieces of evidence.

In addition, having the intervention rather than the study as the unit of analysis is problematic as different study designs address different questions about an intervention. Moreover, as mentioned earlier, no quality appraisal of studies has been undertaken, making it impossible to decide which study reporting on an intervention would be better to include over another.

The analysis of the available evidence in this report includes investigations into various areas of interest (e.g., countries with more evidence; evidence gaps; the prevalence of evidence by sub‐regions—South Asia, Pacific, East Asia; the prevalence of evidence by service setting, etc.) as well as, looking into the different types of intervention and outcome foci. Results of the analyses are discussed in the ‘Results’ section.

### 4.8 Data collection and analysis

#### 4.8.1 Screening and study selection

##### Title and abstract screening

All search records have been screened against inclusion and exclusion criteria. During this first round of screening, two reviewers independently looked at the titles and abstracts and only those deemed relevant to the topic made it to the next round of full‐text screening.

##### Full text screening

The full text for the studies which were included if studies were eligible at the title and abstract screening stage, were screened by two reviewers independently against the inclusion and exclusion criteria. At the end of this step, only studies which are to be included in the EGM remained and data was extracted and charted from these.

The entire search process and the screening outcomes have been documented using a PRISMA Flow Diagram (Moher et al., 2009) so that the readers are able to follow, and potentially replicate, all steps of the review process (see ‘Results’ section).

#### 4.8.2 Data extraction and management

The data extraction process involved gathering information about:

- The study (document/report) title, year, author(s)
- The aim, brief description, content, and length of each intervention/study
- The setting (early years, mainstream school, or special school) and country
- Target population and sample size
- The intended professional development outcomes/and any unintended outcomes
- The results/effectiveness data (i.e., information about program effectiveness if provided)

At least two reviewers independently extracted data from each study and resolved any differences through consultations. This involved in‐depth discussion of the study and the inclusion/exclusion criteria until agreement was reached. Any contextual information about the reason for an intervention or descriptive information about how it had achieved its outcomes were also recorded. The entire data extraction process was managed using MS Excel. See data extraction Table A2.

#### 4.8.3 Tools for assessing risk of bias/study quality of included reviews

In line with the Campbell EGM guidance that critical appraisal of all included studies is desirable but not mandatory (Saran & White, 2018; White et al., 2020), a decision has been made to exclude this step in the current EGM as the timeframe for this EGM is shorter than a full‐sized systematic review (Ahmed et al., 2020, p. 6).

While the research team understands that undertaking a critical appraisal of evidence quality is a key component for any review, as this EGM is diverse nature and covers many different types of evidence, it was not possible to identify an appraisal framework or tool which allowed for differentiated evaluations of the of the different qualitative and quantitative methodological designs. Some researchers have established that applying an appraisal criterion in a universal way can be quite problematic, particularly in the case of qualitative research, as it is highly dependent on a researcher's ability to judge the evidence in predictable and established ways, irrespective of its aims and purposes (Smith, & McGannon, 2018). Researchers have also argued that currently available checklist and framework appraisal methods for qualitative research apply an overall ‘approach to “qualitative” research without sufficiently differentiating between the different methodological approaches (e.g., Grounded Theory, Interpretative Phenomenology, Discourse Analysis) or different methods of data collection (interviewing, focus groups and observations)’ (Williams et al., 2020; p. 10). Additionally, in the LMICs where attempts to collect evidence are already scarce, in the absence of tailored, method‐specific appraisal tools for qualitative designs (Rolfe, 2006; Williams et al., 2020), a quality appraisal using checklists or frameworks could potentially contribute to poor uptake and use of research, even if such research insights have a key role to play in informing evidence‐based decision making.

In this context, it should also be noted that the research team attempted to use some of the available quality appraisal tools which seemed to suit the evidence materials found in this EGM (such as the Critical Appraisal Skills Programme (CASP), 2018; the Joanna Briggs Institute (JBI) Critical Appraisal Checklist, 2017; and the Quality In Prognosis Studies (QIPS) tool—Hayden et al., 2013). However, the team concluded that any ratings based on these tools would provide a false sense of comparability of the very diverse materials and of the reported results. Hence, it was decided not to proceed with a quality appraisal as already foreshadowed in the protocol of this study (Ahmed et al., 2020, p. 6). Still, the desirability of having quality appraisal tools appropriate for the diversity of materials frequently found in EGMs is reflected in recent work to develop tools which are more suitable for this purpose (Mader et al., 2022).

**Figure 7:**
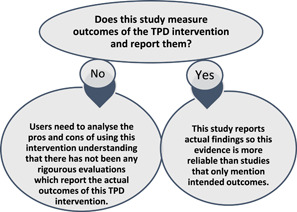
Steps for providing guidance about using evidence from this EGM. EGM, evidence and gap map.

Therefore, instead of undertaking a quality appraisal on the individual studies, the research team has provided some guidance for interpreting the status of outcomes depicted on this map, purely based on the differences between studies that reports actual outcomes versus those that only report intended outcomes (see Figure 7). This is to ensure that users of this EGM do not deem all evidence included here to be of equal strength and magnitude. While the intervention discussed in a study may be a good one, but the team wants to emphasise this distinction between studies which collected outcome data and reports that as opposed to those interventions which promise to do many things but has no data available to prove the claims.

#### 4.8.4 Methods for mapping

The EGM was created using common web development languages such as HTML, CSS, JavaScript and d3.js. The EGM includes responsive design elements for example, the layout and functionality adapt according to the screen size of the user's device. All included studies and their characteristics (see Table A2) were extracted from the MS Excel file that was used for the data extraction process and uploaded to the ACER server in.csv file format. The interactive, online EGM is hosted on the ACER data visualisation site.

## 5 RESULTS

This section covers the following: Description of the studies, Synthesis of included studies, Discussion of the results, Potential biases in the mapping process, and Conclusions.

### 5.1 Description of studies

#### 5.1.1 Results of the search

**Figure 8:**
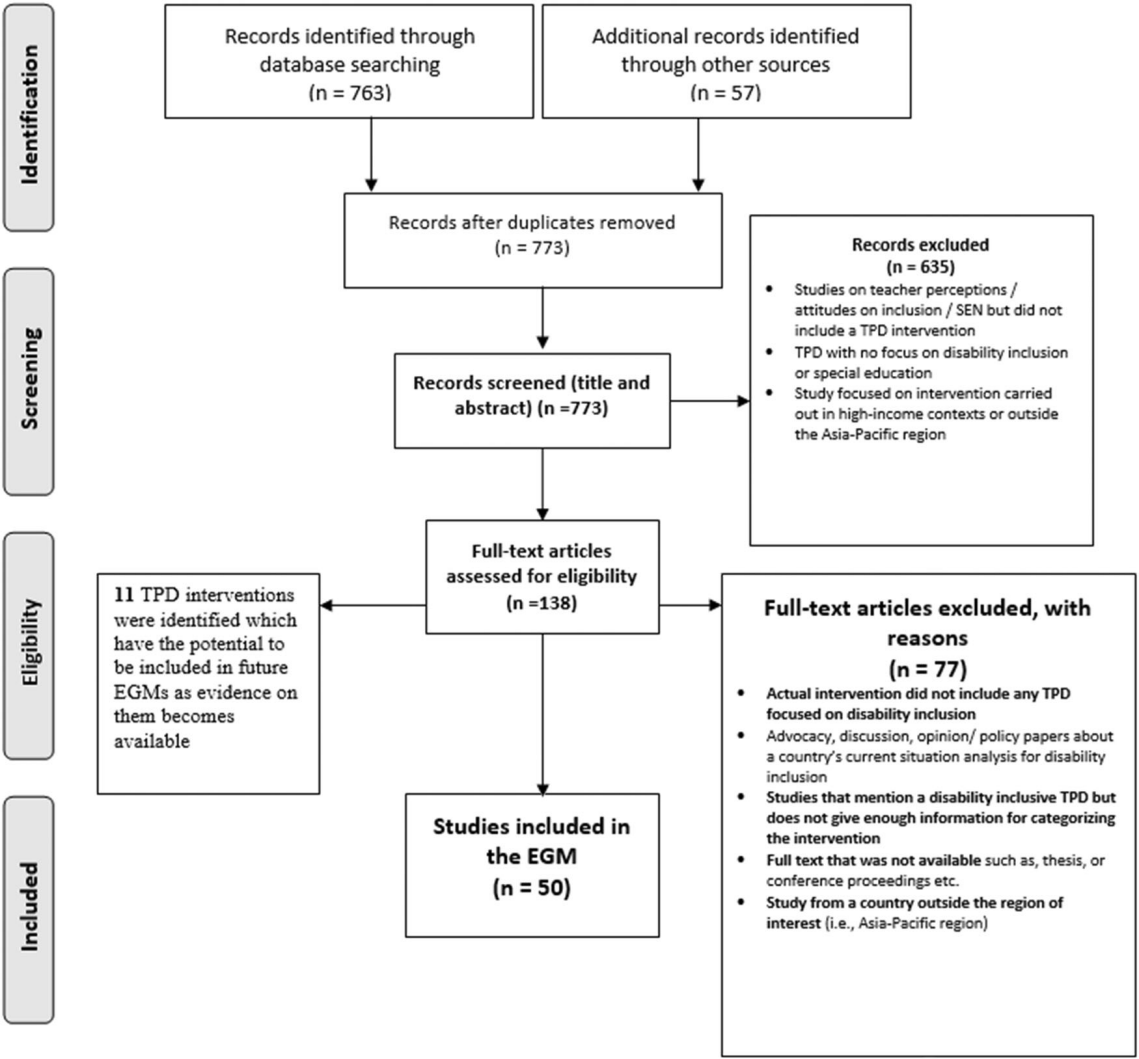
PRISMA Flow chart from Moher et al. (2009)

A PRISMA flow chart presents the results of the search (Figure 8).

#### 5.1.2 Excluded studies

The six characteristics of excluded studies are provided below:

- Discussion/advocacy papers and policy documents
- Does not include any TPD intervention
- Not focused on education for the inclusion of students with a disability
- Not enough information provided about the intervention/not available
- Not in the Asia‐Pacific region
- Duplicates

The following is an example of an excluded study (Sucuoğlu et al., 2015):

The Preschool Inclusion Program (PIP) was developed as a part of The First Inclusive Preschool Project (FIPEPT) in **Turkey** granted by the Scientific and Technological Research Council of Turkey. The aim of this study was to evaluate the effects of this in‐service teacher training program on teacher outcomes. The teachers’ knowledge and attitudes regarding inclusion, classroom management strategies, and their relationships with children both with and without disabilities were evaluated using self‐report instruments. The training supported the teachers to use evidence‐based and developmentally appropriate strategies in their classrooms. Topics covered (a) providing information related to the learning characteristics of children with special needs and inclusive practices; (b) assessing the developmental performance of children with disabilities and writing an IEP; (c)adapting and modifying the preschool curriculum for SWD; (d) supporting language and communication skills; (e) using naturalistic teaching strategies; (f) managing inclusive classrooms (through proactive strategies and functional behaviour analysis) and supporting children with challenging behaviours; and g) engaging and partnering with the families of children. The program evaluated the effects of the PIP on teacher‐level outcomes and reported that the program improved teachers' knowledge, attitudes, and many components of classroom management, as well as increased teachers ‘closeness’ with their students and declined ‘conflicts’. The teachers were highly satisfied with the training and felt very well supported.

Although this intervention is a good example of a TPD on education for the inclusion of students with a disability, the program was conducted in Turkey which is outside this EGM's area of geographical focus, and therefore was excluded.

#### 5.1.3 Studies awaiting classification (if applicable)

See Supporting Information: Appendix B for details

### 5.2 Synthesis of included studies

#### 5.2.1 Types of evidence

This EGM includes evidence from two different sources: systematic database searches and practice‐based evidence from implementation research (Table 2). A definition for each of these terms used is also included in Table 2. Evidence varies from descriptive program documents that did not include systematic analysis of the extent to which the programs attained their intended outcomes to studies which followed an experimental design with a random allocation to treatment and control groups. Many studies did not report information about the teacher sample size (*n* = 24) while a few (e.g., Sagun‐Ongtangco et al., 2021; Simpson et al., 2016) had very small sample sizes (e.g., 3 teachers).

The EGM also includes studies with varying designs: qualitative (*n* = 21), quasi‐experimental (*n* = 10), mixed‐method (*n* = 6) and experimental (*n* = 2). The qualitative studies mainly used observational methods such as interviews, direct observations and focus groups (Table 3).

**Table 3:** Types of evidence by study design

Study design	Number of studies	Count by evidence source
Qualitative methods (interviews, focus groups, reflective journals, direct observations)	21	12 systematic, 9 practice‐based
Quasi‐experimental design (pre–post tests, with or without controls)	10	9 systematic, 1 practice‐based
Mixed‐method design	6	3 systematic, 3 practice‐based
Experimental design (random allocation to treatment control groups)	2	2 systematic
Could not be classified	11	11 practice‐based

The EGM also includes 11 practice‐based studies in which the study design was unclear and thus could not be classified (Table 4).

**Table 4:** Studies with study design that could not be classified

Year	Author	Report title
2020	Global Education Monitoring Report Team, Oxfam India	Inclusive Education in India: Background paper prepared for the 2020 Global Education Monitoring Report.
2021	Grimes et al.	Disability‐Inclusive Education Practices in Afghanistan
2021	Grimes et al.	Disability‐Inclusive Education Practices in Bhutan
2021	Grimes et al.	Disability‐Inclusive Education Practices in India
2021	Grimes et al.	Disability‐Inclusive Education Practices in Maldives
2021	Grimes et al.	Disability‐Inclusive Education Practices in Nepal
2021	Grimes et al.	Disability‐Inclusive Education Practices in Pakistan
2021	Grimes et al.	Disability‐Inclusive Education Practices in Sri Lanka
2018	International Assistance Mission (IAM)	2018 Annual Report.
2009	UNESCO International Bureau of Education & UNESCO Office Jakarta and Regional Bureau for Science in Asia and the Pacific	Malaysia: national report on the provision of inclusive quality primary and junior secondary education for children with disabilities
2018	World Vision International in Laos (WVI‐L)	Lessons Learned Disability Inclusion in Primary Education

### 5.3 Status of outcomes—Intended versus actual

**Figure 9:**
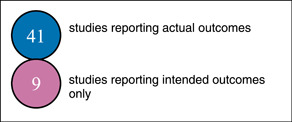
Studies reporting intended versus actual outcomes

In reference to the discussion in section ‘Tools for assessing risk of bias/study quality of included reviews’, the differences in reporting of evidence have been explained in Figure 9. In the EGM, this is shown in two colours—‘blue’ which represents studies that report actual outcomes (*n* = 41), and ‘pink’ representing studies where only intended outcomes are reported (*n* = 9).

It should also be noted that not all studies reporting actual results can be deemed to be of equal strength/quality either. Some studies (Hussein & Vostanis, 2013; Kantavong & Sivabaedya, 2010; Locharoenrat, 2019; Omar et al., 2018) followed rigorous study designs (such as experimental designs with randomised control trials (RCTs) or quasi‐experimental designs) and reported data on the effectiveness of interventions. Others only discussed intended outcomes such as teachers’ perceptions of improvements or asserted that changes had occurred because of the interventions (e.g., Oxfam India, 2020; Grimes et al., 2021a, 2021b).

However, while RCTs are the gold standards for evaluating program effectiveness, effect sizes do not necessarily provide information to the policy makers on how an intervention might be replicated in their specific context, or if it will produce the similar outcomes when the intervention is rolled out across a different setting (Moore et al., 2014).

### 5.4 Aggregate map of evidence gaps

**Figure 10:**
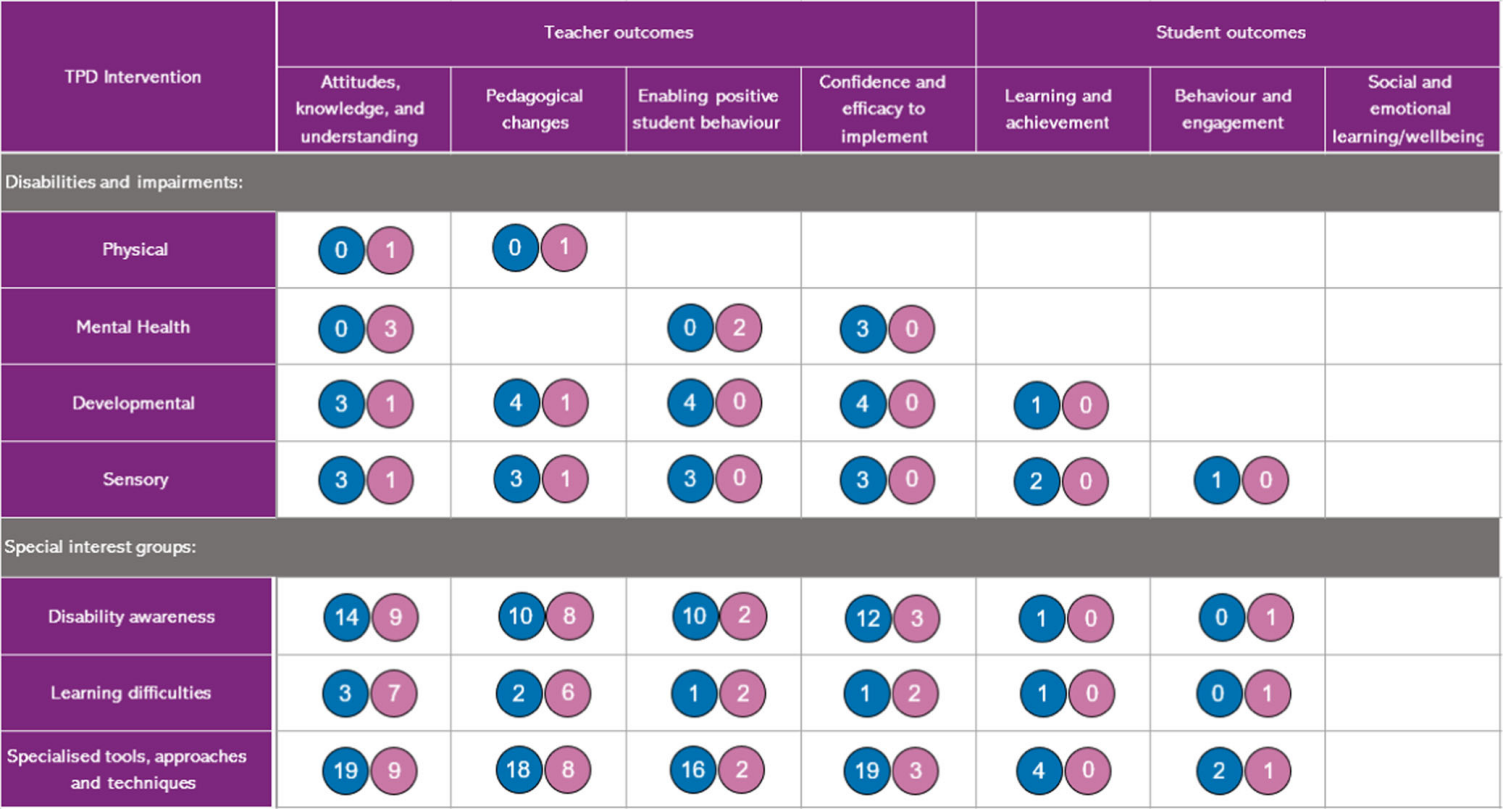
EGM showing studies aggregated by intervention and outcome intersection by evidence type. EGM, evidence and gap map.

The number of studies by intervention and outcome category are presented in Figure 10, this data is split by the status of evidence reporting (i.e., blue bubble depicting actual reporting of outcomes while pink bubble depicts the reporting of intended outcomes only). The figure shows, that the category ‘Disability awareness’, and ‘Specialised tools, approaches and techniques’ are the most frequently found interventions in the included studies. These interventions are mostly focussed on improving the teacher outcomes such as *teacher attitudes, pedagogy, and teacher confidence*. To a lesser extent, these interventions also aim to provide *teachers’ strategies to improve student behaviour*.

Other notable results from the EGM are the dearth of studies that include student level outcomes. This is most prevalent for TPD intervention categories included under the ‘Disabilities and impairments’ such as physical, mental health, developmental and sensory focused interventions.

Another key finding includes the lack of interventions that reported outcomes about students’ *social and emotional learning/wellbeing*.

### 5.5 Intervention setting and location

Around half of the 50 included studies took place in Mainstream schools (24). Early years (8), Special (11) and studies which occurred in Mainstream and Special schools (7) comprised the remaining 26 studies as shown in Table 5.

**Table 5:** Number of studies by setting

Setting	Number of studies
Early years	8
Mainstream	24
Special	11
Mainstream and Special	7

The distribution of studies by country is shown in Table 6. Of the total of 50 studies from 16 countries, the largest number of studies was found in Thailand (7), followed by China, India, and Vietnam (5 each).

**Table 6:** Number of studies by country

LMICs in the Asia‐Pacific region	Number of studies
Afghanistan	2
Bhutan	1
China	5
Fiji	2
India	5
Indonesia	3
Lao PDR	4
Malaysia	3
Maldives	1
Nepal	3
Pakistan	2
Philippines	4
Sri Lanka	2
Thailand	7
Turkmenistan	1
Vietnam	5

Abbreviation: LMIC, low‐ and middle‐income country.

Evidence of teacher professional learning for inclusion or working with children with disabilities was found in only 16 of the 41 LMICs listed in DFAT's list of LMICs in the Asia‐Pacific region (DFAT, 2018). In other words, no evidence could be found in 25 LMICs in this region. Having evidence in less than half the countries clearly demonstrate the need for more research on this topic in the LMICs of the Asia‐Pacific region.

While there may be other professional development initiatives that have taken place or are in progress in this region, they are either not published as research studies or practice‐based evidence and/or did not match the inclusion/exclusion criteria for this review.

Table A2 provides details of the included studies, including key characteristics of the interventions.

### 5.6 Intervention categories and their intended outcomes

To be included in this EGM, all studies had to demonstrate an intention of impacting teachers’ knowledge, attitudes, behaviours, and practice, while a few studies also presented a few expected outcomes for the students –the term intended outcomes is used for these (Table 7).

**Table 7:** Categories of included studies by intended outcomes—Teacher level

Intended outcomes	Description	Number of studies
Attitudes, knowledge, and understanding of education for the inclusion of students with a disability	Relates to teachers’ attitudes (acceptance, confidence, and self‐ efficacy) towards students with disabilities and knowledge about and understanding of inclusive practices.	37
Pedagogical changes	Focuses on teachers’ gaining skills to improve pedagogical practice for the purpose of improving inclusive practices and educational outcomes for children with disabilities.	30
Enabling positive student behaviour	Enables teachers to develop strategies to manage behaviour issues more effectively among students with disabilities.	25
Confidence and efficacy to implement inclusion	Relates to teachers’ confidence, and self‐efficacy for implementing disability inclusive approaches/strategies.	29

The intended outcomes for teachers for the studies included in this EGM are improvements in teacher knowledge, understanding, attitudes, teaching techniques, behaviour management skills, confidence, and self‐efficacy (Table 8). Earlier research suggests a positive correlation between the attitudes of teachers towards SWD through their perceptions of knowledge of policies and procedures and instructional strategies (Alfaro et al., 2015).

**Table 8:** Categories of included studies by intended outcomes—Student level

Intended outcomes	Description	Number of studies
Learning and achievement	Results in positive learning and achievement outcomes for the students.	4
Behaviour and engagement	Changes in students’ behaviour and engagement.	3
Social and emotional learning/wellbeing	Changes to the way students thinks of/feels about themself and of others (their peers), leading to changes in self‐esteem, learning capacity and the sense of school‐belonging.	0

Exploration of the interventions from the included studies indicates that, compared with the large number of studies which intended to improve teacher outcomes, far fewer studies tried to also have an impact on student outcomes (Table 8).

A few studies had multiple intended outcomes for both teachers and students which explains why the mapping of the outcomes according to these categories added up to a larger number of studies than the total of 50 included studies.

### 5.7 Reporting of study outcomes

Overall, 41 of the included studies report ‘actual’ program outcomes. The term ‘actual’ refers to any changes which were intentional or unintentional that were seen because of the TPD intervention. While a closer look at the reported actual outcomes for the included studies show that 31 studies provide evidence that the included TPDs contribute in some way to improving teachers/educators’ attitudes, knowledge and understanding on issues around disability and inclusion. This category is by far the largest as most interventions for TPD are focused on disability & inclusion awareness. Fifteen studies discuss interventions contributing to improvements in instructional practices in classrooms, while fourteen studies report outcomes for enabling positive student behaviour. Another nine studies reported improvements in teachers’ confidence to deliver inclusive approaches and strategies in the classroom. See Table A2 for details on reported outcomes.

However, where studies did report some student‐level actual outcomes, these mainly revolved around student's learning or achievement scores, classroom behaviours and engagement in classrooms, and their social and emotional learning or wellbeing.

Some of the key outcomes from the included studies that reports on student‐level outcomes are discussed next.

**Improved students’ learning and achievement**. Four of the included studies (Banerjee et al., 2016; Kantavong & Sivabaedya, 2010; Martin et al., 2001; STS, 2017) report improvements in student achievement (including test scores) to demonstrate the effect of the program on students.

**Encouraged positive classroom behaviour and student engagement**. Five included studies report improvements in student behaviours and engagement mostly through observational data collection (Muttiah et al., 2018; Opartkiattikul et al., 2016; Grimes, 2021; HEART, 2013; Owen, 2019). The teachers in these interventions claim to have successfully applied their learning in their classrooms which could be seen in their students’ attention and participation.

**Developed social and emotional learning/wellbeing in the students**. Although research indicates that the understanding of social‐emotional competencies is linked to greater student well‐being and better school performance and the failure to achieve this competence can lead to a variety of personal, social, and academic difficulties (Damon et al., 2006; Durlak et al., 2011; Guerra & Bradshaw, 2008), none of the studies started with an intention towards improving social and emotional learning/wellbeing outcomes of the students. Yet only the study by Sagun‐Ongtangco and colleagues (2021) found some positive changes in the students after the intervention in terms of their self‐perceptions and social awareness, but the scope of the impact was unclear.

## 6 DISCUSSION

### 6.1 Summary of main results

The EGM also revealed that a significant number of programs focus on teacher attitudes and understanding of disability. This is quite understandable as many of the countries in the region have only recently started to move towards inclusive education whereby changing teacher attitude and understanding of disabilities is the foundational step. Similarly, this EGM could not identify evidence related to TPD that cover learning assessments for SWD ‐ that is the methods and accommodations required to enable the participation of SWD in assessments. It validates the concern raised by prior research (Chakraborty et al., 2019). Evidence on interventions which support teachers in the use and application of assistive devices is also needed. A critical aspect of teaching students with disabilities and complex needs relates to augmentative and alternative communication (AAC) for students who use technological devices for communication. Professional learning for teachers to support these kinds of technology use is vital for inclusion of SWD, some of whom may be non‐verbal.

### 6.2 Areas of major gaps in the evidence

This EGM on disability inclusion TPD covers interventions undertaken in LMICs in the Asia‐ pacific region over the last two decades. The EGM has found 50 studies on TPD interventions which aim to support disability inclusion. Only three studies report interventions for supporting mental health amongst students with a disability (Hussein & Vostanis, 2013; IAM, 2018; Shah & Kumar, 2012) and one study that reports a TPD intervention related to supporting students with physical disabilities (UNESCO, 2009) where the teachers received training on mobility disability, as part of a larger TPD program. Besides around half (24) of the 50 included studies took place in Mainstream school settings.

As TPD aimed at the inclusion of students with a disability is regarded as having a positive influence on teacher attitudes and knowledge regarding inclusion and children with disabilities to improve student outcomes in line with previous research (Savolainen et al., 2020; Van Mieghem et al., 2020), most of the identified TPD interventions focused primarily on teacher attitudes, awareness and understanding of disability, pedagogies, and confidence building rather than, for example, enabling positive student behaviour. Nevertheless, a few studies covered TPD interventions that aimed to improve the learning outcomes for SWD.

Moreover, none of the studies included in this EGM covered TPD programs designed to support children with disabilities during emergencies/crisis situations, which should be a key focus particularly in light of the current global events such as during pandemics and environmental disasters (Svalina & Ivić, 2020; Tlili et al., 2021).

Furthermore, not one TPD program could be identified that covered training around learning assessments for students with disabilities, such as using different assessment methods and/or special accommodations required to ensure that SWD can participate in learning assessments. This is key gap as assessment for SWD has already been earmarked as being critical for evaluating the learning outcomes of SWD (Chakraborty et al., 2019).

Another key finding was that while numerous research studies demonstrate the importance of Social Emotional Learning (SEL) skill development for teachers (Benn et al., 2012; Durlak et al., 2011; Jennings et al., 2013; Jennings et al., 2017; Jones et al., 2013; Roeser et al., 2013) and particularly for teachers supporting SWD (Alvarez, 2007; Jennings & Frank, 2015; Jones et al., 2013); this EGM did not find any TPD intervention that focused primarily on supporting teachers in this area.

### 6.3 Overall completeness and applicability of evidence

It should also be noted that the EGM did not find any evidence of disability inclusive TPD in 25 (out of 41) LMICs in the Asia‐Pacific region, even after including evidence that are practice‐based. There are also another 11 interventions which are presently ongoing in this space and could have enough data available soon to be included in future evidence synthesis. Supporting Information: Appendix B provides a list of these programs. So, while there may be other TPD initiatives that have taken place or are in progress in the LMICs in this region, these have not published enough evidence which could be picked up through the systematic searches or match the inclusion/exclusion criteria for this EGM.

In terms of the applicability of these findings, in recent times there has been substantial reform efforts across education sectors in LMICs in the Asia‐Pacific region, in line with Article 24 which is focused on the education of children with disabilities (CRPD, 2016). Yet, as this EGM can attest, not much research has been undertaken into teachers’ professional development, readiness, and adaptation as educators for creating inclusive learning experiences for students.

### 6.4 Potential biases in the mapping process

Eligible studies or evidence were restricted to those published from 2000 to the start of 2022 and published in English only.

The search for ‘grey’ literature has been challenging because of the large number of potential sources (e.g., each development partner has its own database) with keyword searches bringing up many activities. While some of the main sources (e.g., ADB) were searched, all programmes or studies could not be covered due to time limitations and mostly the first three to five pages of the search results were screened for inclusion. Consequently, some eligible studies may have been missed.

Another difficulty stems from the categorisation of interventions. Education for the inclusion of students with a disability is a complex topic and there can be overlaps in interventions which focus more broadly on the inclusion of all children encompassing those from minority communities, remote, rural settings, or internally displaced populations. This issue has been dealt with based on expert consultations and the available information to mitigate this issue as far as possible.

Although the World Health Organisation recognises depression, bipolar affective disorder, anxiety disorders, dementia, substance use disorders, intellectual disabilities, and developmental and behavioural disorders with their onset typically occurring in childhood and adolescence as mental health impairments (WHO, 2013) much of the research literature in inclusive education (as reviewed in this EGM) did not include interventions for students with many mental health difficulties, which are often addressed within the mental health literature. The research team has tried to include most of these initiatives by hand searching. Yet, some mental health initiatives focused on professional learning could have been missed due to the review's search strategy.

This EGM was focused on the Asia‐Pacific region as a strategic priority for DFAT, ACER's partner in the ACER‐GEM centre. As a result, professional learning interventions in other regions, particularly in Africa, which support education during crisis/epidemics—for example, during Ebola virus and Human Immunodeficiency Virus (HIV) outbreaks—were excluded from this review.

### 6.5 Strengths

This EGM provides up‐to‐date information on interventions for TPD for disability inclusion. It covers 50 studies/papers published between January 2000 and December 2021, of which a majority (*n* = 29) have been published in the last 5 years. There has not been any other EGM which focuses solely on TPD for disability inclusion. Therefore, this EGM can assist funders and implementing agencies when making decisions as to how to better support LMICs in the Asia‐ Pacific region to reach the following SDG targets (UNESCO, 2016):

- Developing quality teachers for the global inclusive education agenda (SDG 4.c)
- By 2030, ensure that all girls and boys complete free, equitable and quality primary and secondary education leading to relevant and effective learning outcomes (SDG 4.1)
- By 2030, eliminate gender disparities in education and ensure equal access to all levels of education and vocational training for the vulnerable, including persons with disabilities, indigenous peoples and children in vulnerable situations (SDG 4.5).

It is noteworthy that all included studies and reports identified through systematic and wider searches were screened and coded by at least two reviewers, which improves confidence in the evidence synthesis methods.

### 6.6 Limitations

Although the EGM followed a comprehensive search strategy using predefined eligibility criteria, inevitably there are limitations to our approach.

Research indicates systematic reviews and impact evaluations which report robust results about program effectiveness and can be viewed as higher quality evidence. However, most of the primary studies and evidence materials included in this EGM are from observational studies and program reports and therefore only a few included effectiveness data.

Some implementation research evidence, or other practice‐based program documents were excluded because they did not meet the strict inclusion criteria for this EGM. We have referred to these in the section ‘Studies awaiting classification’. These need to be reviewed in the future and any emerging reports about these interventions could be included in future EGM's.

Focusing on low‐ and middle‐income countries also meant that evidence from programs being undertaken in high income education systems such as Singapore, Hong‐Kong, New Zealand, and Australia were excluded. The aim of this review is to capture the scenario and evidence gaps in LMICs in the Asia‐Pacific region to encourage further uptake of interventions and research in these countries.

### 6.7 Stakeholder engagement throughout the EGM process

The EGM is also unique in its participatory approach in protocol development involving key regional development partners and implementing agencies, such as DFAT and CBM, as well as several rounds of feedback from regional research organisations that operate in this space, for e.g., the Research in Inclusive & Specialised Education (RISE).

## 7 AUTHORS' CONCLUSIONS

This report illustrates the critical value of evaluating and publishing evidence from disability inclusive TPD interventions in LMICs. More rigorous studies with larger sample sizes and higher evidence quality that report effectiveness data particularly about outcomes for children with disability are still required. There is also a gap around publishing program effectiveness data for interventions that have been conducted in the LMICs of the region. Therefore, undertaking and publishing results from impact evaluations for practice‐based interventions could strengthen the evidence‐base in these LMICs.

The EGM provides access to existing evidence to ensure that decision makers are aware of the available programs and their performance, before investing funding and resources into new TPD programs to help education systems reach their target of developing quality teachers for the United Nations (UN) global inclusive education agenda (target SDG 4.c).

### 7.1 Implications for research, practice and/or policy

For any educational setting to be effective in including all children efforts must include partnerships with and involvement from teachers, staff, parents, and the school community.

Education in the Asia‐Pacific is undergoing a transformation from segregation to integration to inclusion. In such a situation, teachers in special education schools have the potential to support mainstream teachers for adopting inclusive practices. ‘In a dual system, special schools often provide additional support in the transition of students with disabilities from segregated to general schools’ (Chakraborty et al., 2019).

In a majority of LMICs, teachers may not be part of a training program or apply inclusive instructional methods, which is further amplified by large differences in nation‐wide standards for teacher training from country to country (Hayes & Bulat, 2017).

Evidence from this EGM can help policymakers dive deeper into the TPD interventions in this area and understand how these types of professional learning for teachers can contribute to changes in teacher attitudes and knowledge about inclusion and children with disabilities, and ultimately influence student‐level outcomes. However, more rigorous program evaluation and impact measurement is needed to understand the full extent of impact of disability inclusive TPD on the outcomes for children with disabilities.

The lack of evidence regarding in‐service TPD for supporting children with disabilities during emergencies also indicates the need for policymakers to quickly focus on developing interventions for such events. This particularly applies given the added challenges for children with disabilities during the recent pandemic, which are expected to take more children out of schools than ever before (McClain‐Nhlapo, 2020). The effects of this interruption to children's schooling will need to be considered on top of any other disaster risks the countries in the region face frequently.

Several implications of this EGM are discussed as follows:

- More primary research in LMICs in the Asia‐Pacific region is required around TPD interventions aimed at education for the inclusion of students with a disability. Experimental studies involving some form of pre–post‐test or intervention‐control group design, which are more valuable for evaluating the effectiveness of programs in terms of their desired outcomes, are needed to ensure higher confidence in the evidence collected.
- Smaller Pacific Island countries may benefit from such further research. This may be facilitated by forming a consortium of partners through an evidence hub that collates evidence on program effectiveness, keeping in mind that the model of education for the inclusion of students with a disability vary across the islands. A recommendation would be to use an existing structure, such as the Pacific Data Hub that is already working to collect data within the region. DPs can partner with them to conduct program evaluations that are robust and ensure these are published through appropriate channels, as undertaking separate evaluations of programs in each small island state can be expensive and time consuming.
- A key observation from this EGM is that while the World Health Organisation recognises mental health difficulties as psychosocial disabilities (WHO, 2013) most of the research literature in disability inclusive TPD did not include much work on teachers’ learning for supporting student mental health and psychosocial wellbeing. These studies are potentially included in the mental health literature. Therefore, further research into school mental‐health and psychosocial wellbeing in the context of education for the inclusion of students with a disability is recommended to identify such TPD initiatives.

Where possible, future research also needs to keep in mind the impact of the pandemics and other natural disasters, which are quite common in the region, and focus on ways to support SWD during crises.

## CONTRIBUTIONS OF AUTHORS

Working closely with the GEM Centre, this EGM has been undertaken by a team from the Australian Council *for* Educational Research (ACER) led by **Ms. Syeda Kashfee Ahmed**. Ahmed has been trained through The Centre for Evidence‐based Practice South Australia (CEPSA): A Joanna Briggs Institute Centre of Excellence. Ahmed has developed and co‐ordinated the EGM team, discussed and assigned roles for individual team members, liaised with the editorial team and has taken responsibility for the on‐going updates of this EGM.

The core review team also includes **Dr. David Jeffries, Ms. Anannya Chakraborty, Mr. Toby Carslake**, and **Dr. Petra Lietz**. The core team members were primarily responsible for the key review tasks, including eligibility screening, quality assessment, coding of studies, data extraction, development of the online interactive EGM and writing of the review report.

Other team members, Ms. Budiarti Rahayu (ACER Indonesia) and Dr. David Armstrong (RMIT University) have contributed to the development of the conceptual framework for this EGM and to this descriptive report.

The review team has received extensive feedback and expert advice from Mr. Amit Kaushik (ACER India) and Ms. Kris Sundarsagar (ACER Malaysia) particularly around knowledge of key regional issues for in‐service TPD.

Three of the authors have previously completed a scoping review on young children's learning in economically developing countries (Jackson et al., 2019) while two of the authors have contributed to a recent systematic review on school mental health interventions (Dix et al., 2019). Ms. Ahmed has also contributed to papers in teacher professional learning and development, including Survey of Teachers in Pre‐primary Education (STEPP): Lessons from the implementation of the pilot study and field trial of international survey instruments (Ahmed et al., 2020); Dr. Lietz was a co‐author of several systematic reviews (Best et al., 2013; Lietz et al., 2017) and meta‐analyses (Lietz, 2006) demonstrating her expertise with these methods. Ms. Chakraborty (Chakraborty et al., 2019) has recently contributed to a NEQMAP review on assessments for students with disabilities in the Asia‐Pacific region. Dr. Armstrong, who leads pre‐service teacher education about inclusion and disability, at RMIT University, Melbourne has contributed extensively to the field (Armstrong, 2018; Armstrong & Armstrong, 2021; Armstrong et al., 2015). He provides expert advice to Amnesty International, Parliamentary Inquiries in Australia and to other key stakeholders about enabling educational inclusion and reducing the exclusion of students with disabilities.

## PLANS FOR UPDATING THE EGM

Ms. Ahmed will be responsible for updating this EGM every 5 years, subject to funding availability from the GEM Centre.

## DECLARATIONS OF INTEREST

The authors declare no conflict of interest.

## DIFFERENCES BETWEEN PROTOCOL AND REVIEW

The intervention outcome framework from the protocol^2^ has been revised slightly to reflect the findings. The intervention category ‘multiple impairments and complex needs’ was taken out in the final EGM framework; this was defined in the protocol as:

A more complex form of disability is when an individual has **multiple impairments and complex needs** that is, when two or more conditions simultaneously impact a person's ability to live their life independently. There could be any combination of disabilities impacting someone, for instance a sensory and a physical impairment which causes unique learning needs that cannot be accommodated in a special education setting designed for a specific disorder (AIHW, 2009). There could also be increased complexities from negative attitudes, stereotyping or prejudice by others.

However, teaching students with complex needs was not mentioned by any of the studies captured for this review. Therefore, we have taken this out from our final EGM framework. Instead, we have added a new category under the special interests called ‘disability awareness’ as many interventions included modules/content focused on knowledge about education for the inclusion of students with a disability and intended to create awareness and understanding around the topic.

Some practice‐based interventions were not included because they did not meet the strict inclusion criteria for this EGM. However, these have been included under the ‘Studies awaiting classification’ section of this report. These will be monitored and any emerging reports about these interventions could be included in future EGM's.

Also, while the original plan was to clearly distinguish where evidence is practice‐ based or emerging from ongoing interventions that are selected from grey literature and match the inclusion criteria for this EGM, we decided that this information was better placed in this report. The EGM only shows the status of outcomes reported in two colours—pink and blue—where pink shows that a study that describes an intervention's intended outcomes without providing sufficient evidence of what has been achieved, and blue represents evidence that provide actual results from the interventions. However, not all the actual results can be deemed to be of equal strength/confidence as some studies followed rigorous study designs (such as RCTs, QEs and impact evaluations) and reported effectiveness data from the program. Others merely discussed the teachers’ perceptions of improvements or talked about observed changes, from before and after‐the interventions.

## SOURCES OF SUPPORT

Funded by The Global Education Monitoring (GEM) Centre is a long‐term, strategic partnership between the Australian Council for Educational Research (ACER) and the Australian Government's Department of Foreign Affairs and Trade (DFAT).

## Supporting information

Supporting Information.

Supporting Information.

## Footnotes

1
https://onlinelibrary.wiley.com/doi/10.1002/cl2.1201

2
https://onlinelibrary.wiley.com/doi/10.1002/cl2.1201

## APPENDIX A

### SUMMARY OF FINDINGS TABLES

The outcome categories for the EGM framework are featured in Table A1. The table defines each of the outcome categories and provides examples for studies that will be included in the proposed EGM.

Table A2: Data extraction table

**Table A1:** Outcome categories for the EGM framework

Intended outcomes	Description	Examples
Teachers		
Attitudes, knowledge, and understanding of inclusion and disabilities	Relates to teachers’ attitudes (acceptance, willingness) towards students with disabilities and knowledge about and understanding of inclusive practices	Friendly education to persons with disabilities. A program which provided training for teachers to improve their understanding about persons with disabilities.
		Salim, A., Hidayatullah, M. F., & Nugraheni, P. P. (2019). Investigating effectiveness of disability friendly education training modules in Indonesian schools. *International Journal of Education and Practice*, *7*(3), 286–293.
Pedagogical changes	Focuses on teachers’ gaining skills to improve pedagogical practice for the purpose of improving inclusive practices and educational outcomes for students with disabilities	Transforming Thai Preschool Teachers' Knowledge on Inclusive Practice: A Collaborative Inquiry. A paper which focused on effective teaching techniques for students with disabilities.
		Agbenyega and Klibthong. (2015). Transforming Thai preschool teachers' knowledge on inclusive practice: A collaborative inquiry. *Australian Journal of Teacher Education*, 40(7), 5.
Enabling positive student behaviour	Enables teachers to develop strategies to manage behaviour issues more effectively among students with disabilities	Early Identification of Common Child Mental Health Problems. Focused on training teachers on behaviour management.
		Hussein, S. A., & Vostanis, P. (2013). Teacher training intervention for early identification of common child mental health problems in Pakistan. *Emotional and Behavioural Difficulties*, 18(3), 284–296.
Confidence and efficacy to implement inclusion	Relates to teachers’ confidence, and self‐ efficacy for implementing disability inclusive approaches/strategies	Research conducted for the Kabataang Aralin sa Lahat Ibahagi (KASALI) Project: A study of whether and how children with disabilities are being included in classrooms and early childhood centres. Save the Children. (2018).
		ECCD and elementary school teachers’ testimonies revealed that they benefited from KASALI project. It aided teachers in applying IE instructional strategies efficiently, including teacher's awareness of cultivating learner self‐esteem, and greater confidence in one's capacity.
Students with disabilities		
Learning and achievement	Results in positive learning and achievement outcomes for the students	Professional Learning Program for Enhancing the Competency of Students with Special Needs. Trained teachers to support children with ASD and learning disabilities which led to improvements in students’ reading, spelling and mathematics.
		Kantavong, P., & Sivabaedya, S. (2010). A professional learning program for enhancing the competency of students with special needs. *International Journal of Whole Schooling*, *6*(1), 53–62.
Behaviour and engagement	Changes in students’ behaviour and engagement	Professional Development and Learning by General Teachers Implementing Functional Behavioural Assessment in Thai Inclusive Classrooms. The PD decreased teachers’ aversive approaches and some of them included more preventative and positive approaches with the target students, which in turn led to a decrease in the inappropriate behaviours of the target students during and after the program.
		Opartkiattikul et al. (2015). Professional development and learning by general teachers implementing functional behavioural assessment in Thai inclusive classrooms. *International Journal of Disability, Development and Education*, *6*(5), 545–564.
Social and emotional learning/wellbeing	Changes to the way students thinks of/feels about themself and of others (their peers), leading to changes in self‐esteem, learning capacity and the sense of school‐belonging	Inclusive classrooms: making it work for peers of children with disability. Teacher participation in this inclusive a one‐day workshop where teachers learn about teaching students the ‘Equality and Non‐discrimination Module’ in the inclusive education programme, influenced students’ perspectives of themselves and developed their social awareness skills.
		Sagun‐Ongtangco, K. S., Medallon, K. G., & Tan, A. J. (2021). Inclusive classrooms: making it work for peers of children with disability. *International Journal of Inclusive Education*, *25*(5), 623–639.

**Table A2:** Data extracted from the included evidence

Publication details		TPD intervention description				Study design and data collection		Outcomes			Evidence of impact
First author	Year	Status	Country	Target population	Length/program duration	Study design	Sample size	Intended	Results (reported outcomes and/or effectiveness data if available)	Unintended results (actual)	Reports actual results or impacts (Y/N)
Agbenyega	2015	Completed	Thailand	Preschool teachers	_	Qualitative	11 preschool teachers (7 teachers and 4 teacher assistants) from two government and two private inclusive schools	attitudes	The teachers were able to develop insights into their everyday practices, what they were doing right and what they needed to improve.	_	Y—Changed attitudes
Cheng	2015	Completed	China	Primary and secondary school teachers	4 identical symposia	Qualitative	600 primary and secondary teachers from the Guangzhou China province	attitudes	The primary and secondary teachers understand principles behind assessment and intervention, the importance of language and communication in understanding children's learning challenges and recognise the individuality of each child.	_	Y—Changed attitudes
Eng	2015	Completed	Vietnam	Special educators and paraprofessionals	2 years	Mixed	3 certified and 1 uncertified teacher with 13 teaching assistants in 4 classes. Additionally, 3 physical therapists attended to students with physical therapy needs. 12 staff (who have been employed for more than six months) participated in the evaluation surveys: three teachers (T), six teaching assistants (TA) and three physical therapists (PT).	attitudes	All teachers agreed that they were empowered with skills to facilitate SEN. By the end of this project teachers writing term reports estimated goal achievement rates between seventy and one hundred per cent. Academic goals set for literacy and numeracy experienced greatest success. Behaviour issues had lowest success.	_	Y—Changed attitudes
Hai	2020	Completed	Vietnam	Primary school teachers	2 years	Mixed	20 teachers	Attitudes, pedagogies, enabling	There was increased activity‐based instruction and interaction among teachers and their students, students were exploring new ideas via cooperative learning, there was positive changes in teacher and classmate attitudes and feelings about children with disabilities, and students demonstrated positive attitudes towards all members of a classroom.	_	Y—Changed attitudes, practices, enabling
Hussein	2013	Completed	Pakistan	Primary school teachers	2 days–12 h (6 sessions of about 2 h each)	Quantitative	*n* = 114 teachers: 140 teachers completed the questionnaire pre‐training and 114 completed it post‐training.	Attitudes, confidence	Most teachers (61%) were able to recognise appropriate behavioural management techniques in post‐ compared to pre‐training. The least improvement was reported in relation to causes of mental health problems, as only 2.7% of teachers correctly answered post‐test, compared to pre‐test. In all, 4.16% of teachers were able to give correct responses on the identification of mental health problems and 8.3% on their presentation. The effect size for this analysis was (d=0.43).	_	Y—Changed attitudes, confidence
Kantavong	2010	Completed	Thailand	Primary school teachers	5 days	Quantitative	106 schoolteachers, from 16 schools;	Attitudes, pedagogies, enabling, confidence, achievement	The post–test scores revealed that there was slight improvement in the reading, spelling and mathematical skills of the students taught by the teachers who participated in the program.	_	Y—Student achievement
Klibthong	2014	Completed	Thailand	Educators from preschools/Kindy/ECD centres	3 weeks (15 working days)	Qualitative	16 Thai preschool teachers (4 males and 12 females)	attitudes, enabling, confidence	The program influenced the teachers’ professional knowing, being and becoming. This includes re‐orienting mindsets from tradition, culture, and religion to scientific knowledge, transforming beliefs of incapability and fear to courage and influence, and shifting perspectives from rigid self‐centred practice to inter‐professional networking.	_	Y—Changed attitudes, enabling, confidence
Klibthong	2018	Completed	Thailand	Educators from preschools/Kindy/ECD centres	Workshop—3 days, immersion—3 weeks	Qualitative	16 early childhood teachers (12 female, 4 male)	attitudes, enabling, confidence	The program changed mindsets.	_	Y—Changed attitudes
Locharoenrat	2019	Completed	Thailand	Special educators	Four 1‐h training units	Quantitative	12 special education teachers from a university laboratory school (9 ‐intervention group, 3‐control group)	pedagogies, enabling	The average post‐test score of the trained special education teachers increased to 21.00 (SD = 4.85) [pretest score of the trained special education teachers was 10.61 (SD = 2.74)].	_	Y—Changed pedagogies, enabling
Martin	2001	Completed	China	Special educators	3 h each day, for 3 days	Quantitative	_	Pedagogies, enabling, achievement	Students had gains in reasoning skills as measured by Raven's Standard Progressive Matrices. Other results include improved student engagement, improved teaching techniques (higher level questioning in classroom discussions), and increased use of sign language by teachers.	_	Y—Student achievement, pedagogies, enabling
McCabe	2008	Completed	China	Special educators and other school staff	Ongoing teacher training; initial training for 3 months	Qualitative	The director, the office manager, the director of general services, 4 classroom teachers, 10 assistant teachers and 2 instructional leaders	Pedagogies, enabling, confidence	Teachers were teaching in a more systematic way and were more involved, had better knowledge, and this program model encouraged mentoring and better relationships.	_	Y—Changed pedagogies, enabling, attitudes, confidence
Opartkiattikul	2016	Completed	Thailand	Teachers managing students with behaviour problems	Fortnightly for 3 sessions	Qualitative	4 female teachers with a target student with behaviour problems in their classrooms.	Attitudes, enabling, confidence, behaviours	The teachers used material from the programme and learned very practical skills in a real setting. The teachers decreased their aversive approaches and some of them included more preventative and positive approaches with the target students. The participant teachers also reported that they gained new knowledge and techniques in dealing positively with students who needed behaviour support. In terms of the impact on the students’ most teachers reported a decrease in the inappropriate behaviours of the target students during and after the program.		Y—Changed attitudes, pedagogies, confidence, student behaviours
Muttiah	2018	Completed	Sri Lanka	Special educators	4 instructional sessions (one group training and three individual sessions)—approximately 6 h in total	Quantitative	9 special education teachers	Pedagogies, enabling, confidence, behaviours	All teachers participating in this study showed an increase in the number of evocative communication opportunities provided to students, with some individual variability. The increases ranged from a mean of 9.42 to 16 opportunities during a 10‐min session. The training resulted in increases in number of communication turns taken by students.	_	Y—Changed pedagogies, student behaviours
Opartkiattikul	2015	Completed	Thailand	Primary school teachers	3 sessions over 2 weeks	Qualitative	9 regular classroom teachers (4 from one public school, 5 from one alternative school).	Enabling, confidence	Helped participating teachers acquire basic knowledge and skills in using the FBA process; and provided them with opportunities to practice this new repertoire in their classroom.	_	Y—Changed attitudes, pedagogies
Salim	2019	Completed	Indonesia	Secondary and primary school teachers	2 days	Quantitative	*N* = 30	Attitudes	Helped teachers understand that persons with disabilities need the key aspects of support which are required to create disability friendly education in public schools.	_	Y—Changed attitudes
Shah	2012	Completed	India	Primary school teachers	1 day	Qualitative	1049 teachers from 52 schools	Attitudes, enabling, confidence	Teachers were able to identify children who needed help and referred them to appropriate specialist support.	_	Y—Changed attitudes, enabling, confidence
Simpson	2016	Completed	China	Special educators and other school staff	2 years	Qualitative	The director and 2 teachers from the early intervention site.	Pedagogies, enabling, confidence	The teachers reported that understanding the long‐term and short‐term goals for students helped them plan instruction at the daily, weekly, monthly, and quarterly levels.	The teachers expressed that completing the assessment, developing goals, and gathering data helped to create a more scientific teaching program.	Y—Changed attitudes, pedagogies,
Srivastava	2015	Completed	India	Primary school teachers	4 days	Quantitative	79 regular primary school teachers; control group:41 teachers; experimental group:38 teachers	Attitudes, pedagogies	The teachers demonstrated significant increase in knowledge about teaching methods (biggest effect amongst all outcome variables with ES = 0.54). Positive effects of the training were found on teachers’ attitudes and despite initial neutral attitudes in both groups, the experimental one became more positive as opposed to the control group.	_	Y—Changed attitudes, pedagogies
Xie	2017	Completed	China	Special educators	3 weeks	Quantitative	13 in‐service special educators	Enabling, confidence	The results from surveys suggest a statistically significant gain (before training (*M* = 49.97, *SD* = 5.35) and after (*M* = 54.24, SD = 6.68), *t* (37) = −5.47, *p* < 0.001) in the overall level of self‐efficacy after teacher participants completed HBEIP in‐class training. The teachers felt that they had the knowledge and skills necessary to provide needed supports to young children and their families.	_	Y—Changed attitudes, enabling and confidence
Villa	2003	Completed	Vietnam	Primary school and preschool teachers	_	Qualitative	_	Attitudes, pedagogies, enabling	Teachers reported the training seminars and workshops improved the general quality of the teaching in their schools.	_	Y—Changed attitudes
Yusuf	2017	Completed	Malaysia	Special educators from primary and secondary schools	5 months	Qualitative	12 teachers	Pedagogies	The teacher participants agreed that sharing technology‐based inclusive practices through the pilot study enabled them to improve their professional knowledge through the development and application of reflective and inclusive practices.	A professional learning community that promoted knowledge and development for reflective and inclusive practices was established.	Y—Changed attitudes, pedagogies
Peterson	2018	Completed	Fiji	Special educators and paraprofessionals	_	Qualitative	Teachers from 5 disability inclusion schools	Attitudes, confidence	The teachers indicated (in their interviews) that they have a better understanding of education for the inclusion of students with a disability and were supportive of SWD entering mainstream classrooms.	_	Y—Changed attitudes
Banerjee	2016	Completed	India	Primary school teachers from government primary schools	10 to 50 days	Quantitative	_	Pedagogies, achievement	40 days of active teaching led to large learning gains. In Uttar Pradesh the gains were 0.7 standard deviation in both language and math.	_	Y—Changed pedagogies, student achievement
Kurniawati	2017	Completed	Indonesia	Primary and secondary school teachers without having educational background in special education	32 h of face‐to‐face sessions, and was spread across 4 Saturdays	Quantitative	_	Attitudes, pedagogies, confidence	The training program had a medium to large size effect on teachers’ attitudes and their knowledge about SEN and about teaching strategies. The study did not find a significant effect of the training programme on the behavioural component of attitude and knowledge about dyslexia.	_	Y—Changed attitudes
World Vision	2018	Completed	Lao PDR	Primary and secondary school teachers	5‐day workshop.	Qualitative	71 teachers from 22 primary schools in 22 villages joined a 5‐day workshop on Inclusive Education; 60 teachers wrote Individual Education Plans (IEPs) for 60 students; 19 teachers joined a second workshop on IEPs	Pedagogies, enabling	Most teachers had not been able to set appropriate learning targets and suggested approaches such as ‘sit the child at the front’. No teachers had created learning aids, reportedly due to a lack of time—teachers of multigrade classes particularly felt this pressure. Some teachers had identified a student with a physical or speech impairment but had then written an IEP which did not address these issues at all.	_	Y—Changed attitudes
					Project timeframe 14 months						
Nanwani	2018	Completed	Philippines	Teachers/educators from 12 Early Childhood Care and Development (ECCD) centres and 12 elementary schools.	4 years	Qualitative	6 elementary school classroom teachers and 6 ECCD classroom teachers were observed. 2 elementary school teachers were observed in each of the three cities targeted.	Pedagogies, enabling, confidence	Some promising classroom management practices were observed, but only one of six teachers observed implemented accommodation strategies. The elementary school teachers (comparatively to ECCD teachers) were more aware and responsive to the learning needs of children with disability.	_	Y—Changed attitudes, enabling
Etherton	2003	Completed	Vietnam	Primary and secondary school teachers who had already been trained and were being trained in the 4 provinces	12 years	Qualitative	_	Attitudes	Many teachers seemed to have a substantial paradigm shift in their attitude towards children with disability and the wider social purpose of teaching primary school children.	_	Y—Changed attitudes
Riahta	2018	Completed	Indonesia	Primary school teachers	8 h session over 2 days	Quantitative	26 primary school teachers (74% female and 26% male) in the age group 24–59 years.	Attitudes, confidence	There was no significant change in the attitudes of the teachers.	_	Y—No changes noted
Australian Aid	2015	Completed	Fiji	Teachers/educators and teaching aides of disability inclusive schools	6 years	Mixed	_	Pedagogies, enabling	The program led to increases in enrolment of students with disabilities into mainstream schools.	Trained staff moving to other schools have transferred and applied their newfound skills to the non‐AQEP schools.	Y—Changed attitudes
Save the Children	2018	Completed	Philippines	Primary and preschool teachers	4 years	Mixed	_	Attitudes, pedagogies, enabling, confidence	Trained teachers demonstrated understanding of the concept of inclusive education and implemented inclusive education strategies.	_	Y—Changed attitudes
Owen & Plan International Laos	2019	Completed	Lao PDR	Primary school teachers	_	Qualitative	_	Pedagogies, confidence	Teachers reported that the IEP approach provided some practical steps for teaching children with learning difficulties or children with a disability. Some teachers also identified positive results in student learning in their classroom.	_	Y—Changed pedagogies, student behaviours
School‐to‐school international (STS)	2017	Completed	Philippines	Special education teachers in 15 schools in Luzon, Visayas, and Mindanao,	16 months	Mixed	_	Attitudes, pedagogies, confidence, achievement	The project sensitised teachers to the needs of their students who have low vision or are blind and encouraged them to set high expectations for their students regardless of their visual status. Students in the intervention group had significantly larger gains than their peers in the comparison group on all subtasks on both the Filipino and English EGRAs.	_	Y—Changed attitudes, student achievement
Omar	2018	Completed	Malaysia	Pre‐school teachers from KEMAS Tabika's and Taska's in the Klang Valley	_	Quantitative	_	Attitudes, confidence	The vision screening conducted by pre‐school teachers was effective but would benefit from more training.	_	Y—Enabling
UNICEF	2003	Completed	Nepal	Primary school teachers and special needs educators	_	Qualitative	_	Attitudes, pedagogies, confidence	Teachers had a positive and welcoming attitude towards children with diverse learning needs. Teachers encouraged both children with and without disabilities to participate in class and in group activities. The teacher also allows them to engage in learning activities that they enjoy.	The intervention created an environment of acceptance of disability among non‐disabled peers.	Y—Changed attitudes, pedagogies
Pham	2021	Completed	Vietnam	Primary school teachers	4 days, over 2 weekends	Mixed	Teachers/educators from 4 special education classes and 20 general education classes from Grades One to Five.	Attitudes, enabling, confidence	There was a positive change in teachers’ understanding and level of confidence in using the Function‐based Intervention and supporting students.	_	Y—Changed attitudes, confidence, enabling
Oxfam India	2020	Completed	India	Primary school teachers	2‐3 days or 3 to 5 days	N/A	_	Attitudes, pedagogies, confidence	_	_	N
UNESCO	2009	Completed	Malaysia	Special education teachers	_	N/A	_	Attitudes, pedagogies	_	_	N
HEART: Health and Education advice and Resource Team	2013	Completed	Lao PDR	Primary and secondary school teachers	Several years	Qualitative	_	Attitudes, pedagogies, confidence	Teachers are actively supporting students mostly through key strategies that they have been taught in IE training. Schools where teachers had received IE training or refresher courses in IE relatively recently were more likely to be aware of developments in child centred teaching pedagogy. Children with mild and moderate disabilities were mostly being successfully included in their local schools; their attendance improved, and grade repetition dropped significantly.	Local communities welcomed the project because they could see that all the children were benefitting from improved quality of education.	Y‐ changed attitudes, enabling, confidence, pedagogies, student behaviours
VSO	2021	Ongoing	Nepal	Primary and secondary school teachers who work with girls with disability	Ongoing	Qualitative	100 teachers	Attitudes, pedagogies, confidence	Improved teaching quality and increased inclusive education practice in learning facilities.	_	Y—Changed pedagogies, enabling
Grimes, P and Save the Children, Norway	2009	Completed	Lao PDR	Primary and secondary school teachers	Several years	Qualitative	_	Attitudes, enabling	Teachers are actively supporting students mostly through key strategies that they have been taught in IE training. Children with mild and moderate disabilities are mostly being successfully included in their local schools; their attendance is good and grade repetition has dropped significantly following. Schools which had a close and collaborative working relationship with their local community and parents were far more likely to be successful in developing learner friendly environments.	Schools where teachers had received IE training or refresher courses in IE relatively recently were more likely to be aware of developments in child centred teaching pedagogy.	Y—Changed attitudes, enabling, confidence, pedagogies, student behaviours
International Assistance Mission (IAM)	2018	Completed	Afghanistan	Primary and secondary school teachers	_	N/A	_	Attitudes, enabling, confidence	The Positive Parenting Project in Afghanistan conducted trainings about children's mental health disorders and non‐violent classroom management skills for teachers in schools, as well as supported teachers identify the needs of students with mental health disorders so they can refer them to available services.	Positive changes in communication between the teachers and students in the classes. The teachers are practicing new classroom management skills; none of the teachers use physical punishment anymore.	Y—Changed attitudes, enabling
Munce	2014	Completed	Turkmenistan	Primary and secondary school teachers	Short duration	Qualitative	Teachers from 26 schools (which implemented the CFS program).	Attitudes, pedagogies, enabling, confidence	Teachers reported improvements in knowledge and understanding.	_	Y—Changed attitudes
Sagun‐Ongtangco	2021	Completed	Philippines	Primary and secondary school teachers	1‐day workshop	Qualitative	3 regular schoolteachers.	Attitudes, enabling, confidence	The program improved the teachers’ knowledge, feelings, and values towards inclusion in the process of module implementation. There was also some impact found such as students changed perspectives of themselves, social awareness, and appreciation of the module.	The teachers felt closer to the students.	Y—Changed attitudes, enabling, students SEL
Grimes	2021a	Completed	Sri Lanka	Primary and secondary school teachers; special educators	_	Qualitative	_	Attitudes, pedagogies	_	_	N
Grimes	2021b	Completed	Pakistan	Primary and secondary school teachers; special educators	_	Qualitative	_	Attitudes	_	_	N
Grimes	2021c	Completed	Maldives	Primary and secondary school teachers; special educators	_	Qualitative	_	Attitudes, pedagogies, enabling, confidence, behaviours	_	_	N
Grimes	2021d	Completed	India	Primary and secondary school teachers; special educators	_	Qualitative	_	Attitudes, pedagogies, enabling, confidence	_	_	N
Grimes	2021e	Completed	Bhutan	Primary and secondary school teachers; special educators	_	Qualitative	_	Attitudes, pedagogies	_	_	N
Grimes	2021f	Completed	Afghanistan	Primary and secondary school teachers; special educators	_	Qualitative	_	Attitudes, pedagogies	_	_	N
Grimes	2021g	Completed	Nepal	Primary and secondary school teachers; special educators	_	Qualitative	_	Attitudes, pedagogies	_	_	N
