# Corrigendum

**DOI:** 10.1002/cl2.1315

**Published:** 2023-02-25

The article listed below, intended for publication in Campbell Systematic Reviews, Volume 10, Issue 1, an issue entitled ‘*PROTOCOL: The impact of detention on the health of asylum seekers: A protocol for a systematic review*’ was inadvertently published in the incorrect, volume 13, Issue 1. The cover page of the PDF had the wrong date on it, and the publication date of the protocol says 2 January 2017, which is wrong. The correct publication date is 2 January 2014.

