# Supplementary material for: Teacher professional development for disability inclusion in low‐ and middle‐income Asia‐Pacific countries: An evidence and gap map
Source: Campbell Syst Rev. 2022 Nov 25;18(4):e1287. doi: 10.1002/cl2.1287 (PMC9700054; doi:10.1002/cl2.1287)
Supplement: Supplementary file 1 — Supporting Information. [file CL2-18-e1287-s001.docx]

# Appendices

## Appendix A Link to online interactive EGM

<https://datavis.acer.org/gem/disability-inclusion-TPD>

## Appendix B Studies awaiting classification

In addition to the evidence found from primary studies and practice-based reports or implementation research related documents on the different TPD interventions in the region, some reports collate findings on interventions from different countries or provide program descriptions of current or planned programs. Such evidence is summarised in Table B1. A few of the interventions mentioned in these reports and documents are already discussed under other primary studies that are included in the EGM. Examples of these include the Child-friendly Schools training in Afghanistan, the Access to Quality Education Program (AQEP) in Fiji, the Reading Beyond Sight project in the Philippines, and the Sarva Shiksha Abhiyan program in India. For some of the other programs - such as the ongoing training programs supported by the Japanese International Cooperation Agency (JICA) in Mongolia and Sri Lanka - information about the TPD intervention was insufficient for them to be included in this EGM.

Table B1. Studies awaiting classification (currently being undertaken, i.e., ongoing or planned)

| Authors | Year | Type | Focus | Countries | Setting | Study link |
| --- | --- | --- | --- | --- | --- | --- |
| Amaltas Institute of Human Development | 2019 | Report | Disability Awareness, Mental | Afghanistan, Bangladesh, India, Nepal, Myanmar, Pakistan | Mainstream; Special | <https://www.gov.uk/research-for-development-outputs> |
| CBM | 2018 | Report | Sensory, Tools | India; Vietnam | Special | <https://www.cbm.org/fileadmin/user_upload/Publications/DID_Series_-_Book_3.pdf> |
| DFAT | 2018 | Report | Disability Awareness, Tools, Sensory | Nepal, Fiji | Mainstream; Special | <https://www.dfat.gov.au/development/performance-assessment/aid-evaluation/strategic-evaluations/development-for-all-evaluation> |
| Grimes, P. & Heijnen-Maathuis, E. | 2018 | Report | Tools | Bangladesh, Indonesia | Mainstream | <http://resource-centre-uploads.s3.amazonaws.com/uploads/inclusive_education_cross-country_peer_review_report_final_4_april_2018_002.pdf> |
| Grimes, P., *et al.;* UNICEF Regional Office for South Asia | 2021 | Report | Disability Awareness, Tools, Learning difficulty | Afghanistan, Bhutan, India, Nepal, Maldives, Pakistan and Sri Lanka | Mainstream; Special | <https://www.unicef.org/rosa/media/16976/file/Regional%20Report.pdf> |
| JICA | 2020 | Website program description | Disability Awareness, Tools | Mongolia | Mainstream | <https://www.jica.go.jp/project/english/mongolia/029/outline/index.html> |
| JICA | 2015 | Website program description | Developmental, Tools | Mongolia | Special | <https://www.jica.go.jp/project/english/mongolia/013/outline/index.html> |
| JICA | 2019 | Website program description | Disability Awareness, Tools | Sri Lanka | Mainstream; Special | <https://www.jica.go.jp/project/english/srilanka/009/outline/index.html> |
| Save the Children | 2019 | Report | Disability Awareness, Tools | Bangladesh, Cambodia, China, Indonesia, Kosovo, Myanmar, Philippines, Romania, Vietnam | Mainstream | <https://resourcecentre.savethechildren.net/document/mainstreaming-inclusive-education-sharing-good-practices/> |
| School-to-School International (STS) | 2017 | Report | Sensory, Tools | India, Philippines | Mainstream | <https://allchildrenreading.org/wp-content/uploads/2019/07/STS_Summary-Report-Round-2_4.24.2018_Lo-Res.pdf> |
| UNESCO | 2009 | Report | Disability Awareness, Tools, Sensory, Developmental, Physical | Samoa, Thailand, Vietnam | Mainstream | <https://unesdoc.unesco.org/ark:/48223/pf0000214603> |

## Appendix C Search statement

**SEARCH STATEMENT - ERIC (VIA EBSCO) SEARCH**

**15 July 2020**

Limiters - Date Published: 20000101-20201231

Search modes - Boolean/Phrase

Note:

- GE = LOCATION IDENTIFIERS
- SU=SUBJECT HEADINGS FROM THE THESAURUS
- Terms not proceeded by a field code were searched as a key word so that the search was not limited to any particular field. The keyword search included title and abstract searching.

COPY OF FINAL SEARCH STATEMENT

( GE (Asia OR “Pacific Islands” OR Afghanistan OR Bangladesh OR Bhutan OR Cambodia OR China OR India OR Indonesia OR Kazakhstan OR Korea OR Korea OR Kyrgyzstan OR Lao OR Malaysia OR Maldives OR Mongolia OR Myanmar OR Nepal OR Pakistan OR Philippines OR “Sri Lanka” OR Tajikistan OR Thailand OR “Timor Leste” OR Turkmenistan OR Uzbekistan OR Vietnam OR Cook Islands OR Fiji OR Kiribati OR “Marshall Islands” OR Micronesia OR Nauru OR Niue OR Palau OR Papua OR Samoa OR “Solomon Islands” OR Tokelau OR Tonga OR Tuvalu OR Vanuatu OR Wallis OR Futuna) OR “Pacific Island” OR “Pacific Islands” OR “Asia Pacific” ) AND ( SU Inclusion OR exclus* OR equit* OR inequit* OR SU “Equal Education” OR marginali* OR SU Disadvantaged OR "activity limitation" OR "participation restriction" OR SU "Special Education" OR SU "Special Needs" OR SU "Slow learners" OR "Differently abled" OR retard* OR SU Disabilities OR SU Disability OR SU Disorder* OR SU Impairments OR handicap* OR abnormal* OR SU Discrimination OR harass* OR SU Bullying OR “Student Diversity” OR SU “Mental Health” OR SU “Mental Disorders” OR SU Accessibility OR SU Poverty OR SU “Physical Development” OR SU sensory OR SU “Intellectual Development” OR psychosocial OR hearing OR SU Deafness OR SU Vision OR SU Blindness OR SU Delayed Speech OR SU Developmental OR SU Autism OR ASD OR SU “Asperger Syndrome” OR SU “Learning Problems” ) AND ( SU (“Early Childhood Education” OR “Child Care” OR Caregiver* OR School* OR “Elementary Education” OR “Primary Education” OR “Secondary Education” OR “Elementary Secondary Education” OR “Preschool Education” OR “Kindergarten” OR “Grade 1” OR “Grade 2” OR “Grade 3” OR “Grade 4” OR “Grade 5” OR “Grade 6” OR “Grade 7” OR “Grade 8” OR “Grade 9” OR “Grade 10” OR “Grade 11” OR “Grade 12” OR “Intermediate Grades” OR “Special Education”) OR “K-12” OR “K to 12” ) AND ( SU ("Professional development" OR training OR “teacher education”) )

275 RESULTS

**SEARCH STATEMENT - ERIC SEARCH (via EBSCO)**

5 January 2022

Limiters - Date Published: 2020-2021

Search modes - Boolean/Phrase

**Note:**

GE = LOCATION IDENTIFIERS

SU=SUBJECT HEADINGS FROM THE THESAURUS

**Search Statement**

GE (Asia OR “Pacific Islands” OR Afghanistan OR Bangladesh OR Bhutan OR Cambodia OR China OR India OR Indonesia OR Kazakhstan OR Korea OR Korea OR Kyrgyzstan OR Lao OR Malaysia OR Maldives OR Mongolia OR Myanmar OR Nepal OR Pakistan OR Philippines OR “Sri Lanka” OR Tajikistan OR Thailand OR “Timor Leste” OR Turkmenistan OR Uzbekistan OR Vietnam OR Cook Islands OR Fiji OR Kiribati OR “Marshall Islands” OR Micronesia OR Nauru OR Niue OR Palau OR Papua OR Samoa OR “Solomon Islands” OR Tokelau OR Tonga OR Tuvalu OR Vanuatu OR Wallis OR Futuna) OR “Pacific Island” OR “Pacific Islands” OR “Asia Pacific”

AND

SU Inclusion OR exclus* OR equit* OR inequit* OR SU “Equal Education” OR marginali* OR SU Disadvantaged OR "activity limitation" OR "participation restriction" OR SU "Special Education" OR SU "Special Needs" OR SU "Slow learners" OR "Differently abled" OR retard* OR SU Disabilities OR SU Disability OR SU Disorder* OR SU Impairments OR handicap* OR abnormal* OR SU Discrimination OR harass* OR SU Bullying OR “Student Diversity” OR SU “Mental Health” OR SU “Mental Disorders” OR SU Accessibility OR SU Poverty OR SU “Physical Development” OR SU sensory OR SU “Intellectual Development” OR psychosocial OR hearing OR SU Deafness OR SU Vision OR SU Blindness OR SU Delayed Speech OR SU Developmental OR SU Autism OR ASD OR SU “Asperger Syndrome” OR SU “Learning Problems”

AND

SU (“Early Childhood Education” OR “Child Care” OR Caregiver* OR School* OR “Elementary Education” OR “Primary Education” OR “Secondary Education” OR “Elementary Secondary Education” OR “Preschool Education” OR “Kindergarten” OR “Grade 1” OR “Grade 2” OR “Grade 3” OR “Grade 4” OR “Grade 5” OR “Grade 6” OR “Grade 7” OR “Grade 8” OR “Grade 9” OR “Grade 10” OR “Grade 11” OR “Grade 12” OR “Intermediate Grades” OR “Special Education”) OR “K-12” OR “K to 12”

AND

SU ("Professional development" OR training OR “teacher education”)

**23 RESULTS**

**Minus 2020 results previously provided**

**18 RESULTS TOTAL**
