# Supplementary material for: Teacher professional development for disability inclusion in low‐ and middle‐income Asia‐Pacific countries: An evidence and gap map
Source: Campbell Syst Rev. 2022 Nov 25;18(4):e1287. doi: 10.1002/cl2.1287 (PMC9700054; doi:10.1002/cl2.1287)
Supplement: Supplementary file 2 — Supporting Information. [file CL2-18-e1287-s002.docx]

**Abbreviations and acronyms**

| ACER | Australian Council for Educational Research |
| --- | --- |
| APA | American Psychological Association |
| ASD | Autism Spectrum Disorder |
| ASHA | American Speech-Language-Hearing Association |
| CBM | Christian Blind Mission |
| DFAT | Australian Government Department of Foreign Affairs and Trade (DFAT) |
| CRPD | Convention on the Rights of Persons with Disabilities |
| DD | Developmental Disability |
| EGM | Evidence and Gap Map |
| GEM centre | Global Education Monitoring (GEM) Centre |
| GEM report | Global Education Monitoring Report |
| ID | Intellectual disability |
| IE | Inclusive education |
| INEE | Inter-agency Network for Education in Emergencies |
| LMICs | Low‐ and middle‐income‐countries |
| OECD | Organisation for Economic Co-operation and Development |
| RISE | Research in Inclusive & Specialised Education |
| SDG | Sustainable Development Goal |
| SWD | Students with disability |
| TALIS | Teaching and learning International Survey |
| TPD | Teacher professional development |
| UN | United Nations |
| UNESCO | United Nations Educational, Scientific and Cultural Organization |
| UNICEF | United Nations International Children's Emergency Fund |
